# New Nostocyclophanes from *Nostoc linckia*

**DOI:** 10.3390/md21020101

**Published:** 2023-01-31

**Authors:** Jingqiu Dai, Casey S. Philbin, Clay Wakano, Wesley Y. Yoshida, Philip G. Williams

**Affiliations:** 1 Department of Chemistry, University of Hawaii at Manoa, Honolulu, HI 96822, USA; 2 Department of Chemistry, University of Nevada Reno, Reno, NV 89557, USA; 3 Center for Biomedical Research, The Queen’s Medical Center, Honolulu, HI 96813, USA

**Keywords:** cyanobacteria, *Nostoc linckia*, nostocyclophanes, [7.7]paracyclophanes

## Abstract

Six new nostocyclophanes and four known compounds have been isolated from *Nostoc linckia* (Nostocaceae) cyanobacterial strain UTEX B1932. The new compounds, nostocyclophanes E–J (**1**–**6**), were characterized by NMR and MS techniques. The known compounds were nostocyclophanes B–D, previously isolated from this strain, and dedichloronostocyclophane D. Structural modifications on the new [7.7]paracyclophane analogs **1**–**5**, isolated from the 80% methanol fraction, range from simple changes such as the lack of methylation or halogenation to more unusual modifications such as those seen in nostocyclophane H (**4**), in which the exocyclic alkyl chains are of different length; this is the first time this modification has been observed in this family of natural products. In addition, nostocyclophane J (**6**) is a linear analog in which C-20 is chlorinated in preparation for the presumed enzymatic Friedel–Craft cyclization needed to form the final ring structure, analogous to the biosynthesis of the related cylindrocyclophanes. Nostocyclophane D, dedichloronostocyclophane D, and nostocyclophanes E-J demonstrated moderate to weak growth inhibition against MDA-MB-231 breast cancer cells.

## 1. Introduction

Filamentous cyanobacteria are prolific producers of biologically potent secondary metabolites [1], often with highly unusual structures [2]. One such unique class of compounds is [7.7]paracyclophanes [3] such as cylindrocyclophanes, nostocyclophanes [4], merocyclophanes [5] and carbamidocyclophanes [6] produced by cyanobacteria; compounds whose activities range from weakly cytotoxic [4] to moderately antibacterial (MRSA) [7]. Structurally unusual, even among synthetic compounds, these [7.7]paracyclophanes contain 22-membered rings formed by the head-to-tail dimerization of an alkyl resorcinol intermediate. Labelling studies by Moore and Bobzin [8] in the 1990s established the acetate origin of most of the carbons and led to a proposed biosynthesis that involved C-C bond formation between an sp^2^ and sp^3^ center that linked the monomers by an unknown mechanism using an unknown intermediate [8]. Studies 20 years later by Balskus et al. identified the biosynthetic gene cluster (BGC) for the cylindrocyclophanes based on an HMG-CoA synthase homolog [9,10]. They subsequently determined that ring formation for that series depended on cryptic halogenation of the alkyl chain by a novel halogenase (CylC) and two subsequent stereospecific Friedel–Crafts reactions. Further studies have defined the substrate scope [11] and examined the key enzymatic interactions [12,13].

Several years ago, as part of a larger NIH-funded project to provide known natural products for biological screening, we began reexamining strains within the Patterson/Moore Culture Collection of cyanobacteria at the University of Hawaii. The original investigations into many of these strains in the 80s and 90s led to the discovery of the first examples of several well-known families of cyanobacterial metabolites. Hence, the primary aim of that project was to regrow strains known to produce those specific metabolites. One of those strains was *Nostoc linckia* strain UTEX B1932, from which nostocyclophanes A-D had been discovered; a family distinguished from other cyanobacterial [7.7]paracyclophanes by additional chlorination on the alkyl chain, and a lack of the HMG-CoA derived methyl group. While isolating nostocyclophanes B–D [4], we also isolated dedichloronostocyclophane D originally reported from a sea hare [14] and several additional minor analogs. Here, we report the structures of these new compounds within the nostocyclophane series. The structures of nostocyclophanes E–J (**1**–**6**) were deduced using NMR and MS techniques. Of particular interest is nostocyclophane J (**6**), a linear analog in which one of the “cryptic” halogenations is evident, and nostocyclophane H (**4**), derived from two different alkyl resorcinol intermediates.

## 2. Results and Discussion

A cryostock of UTEX B1932 was scaled up via progressively larger cultures to a final culture volume of 240 L split over 12 × 20 L glass carboys. After 37 days, the cultures were harvested by filtration. The freeze-dried cell mass was extracted thrice, and the combined extract was separated by multiple rounds of chromatography. LCMS and NMR analyses of the resulting fractions showed the 80% MeOH fraction from a C18 flash column contained the nostocyclophanes of interest. HPLC purification of this fraction yielded the known compounds nostocyclophanes B–D [4] that had been targeted for isolation, along with dedichloronostocyclophane D [14] and six new compounds, nostocyclophanes E–J (**1**–**6**).

Nostocyclophane E (**1**) provided a negative HRESIMS ion [M − H]^−^ at *m*/*z* 617.3599. A molecular formula of C_36_H_55_ClO_6_ was deduced, and this formula did not provide a good match to any known [7.7]paracyclophanes in our available database [15]. The similarity of the ^1^H NMR spectrum of **1** to that of nostocyclophane D indicated that **1** belonged to this family of compounds, which had also been isolated from this fraction. The ^1^H and ^13^C NMR spectra (Table 1 and Table 2) of nostocyclophane E were more complex than those of nostocyclophane D, indicating that **1** lacked symmetry, and hence many of the NMR signals were doubled. For example, in the ^1^H NMR spectrum of nostocyclophane E, four phenolic and four aromatic proton signals were present rather than two of each type as observed for nostocyclophane D. A detailed comparison of the ^13^C NMR data for nostocyclophane D and **1** revealed that C-16 was the point of asymmetry in **1** as this was no longer a chlorinated methine resonating around 60 ppm but now was a simple methylene (30.1 ppm). Analysis of the 2D NMR spectra of **1** confirmed that conclusion. Specifically, H-14 displayed HMBC correlations to one methoxy carbon, three sp^2^ carbons, and two aliphatic methylenes (C-15, -16). In contrast, H-1 displayed the expected HMBC correlations to a methoxy group, three sp^2^ carbons (C-24, -21, -27), one aliphatic methylene, and one halogenated carbon (C-3). Evidence of the expected aromatic rings were HMBC correlations from both sets of sp^2^ carbons, e.g., H-10 and H-12 to C-12 and C-10, respectively, and to quaternary carbons C-11, C-12, and C-13. Finally, while no HMBC correlations were visible in our data to prove the C-7/C-8 and C-20/C-21 connections, these connections are required by the number of degrees of unsaturation.

**Figure 1:**
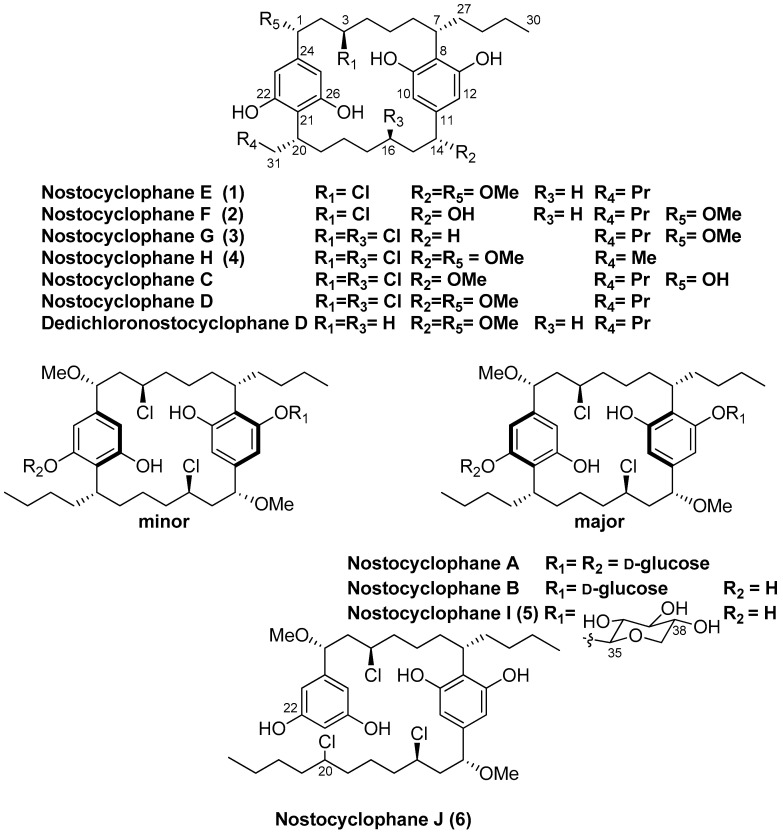
Nostocyclophane structures.

Compounds **2** and **3** were simple analogs of **1** whose structures were readily deduced by comparison of their NMR spectra and MS spectrometric data with the known members of the nostocyclophane structural family (Figure 1). Briefly, nostocyclophane F (**2**) differed from **1** by 14 amu, which could be attributed to a missing methyl group (14-OMe) attached to the corresponding oxygen at C-14. The change in chemical shift of C-14 (73.5 ppm for F vs. 83.6 ppm for E) supports this conclusion. Finally, nostocyclophane G (**3**), with a molecular formula of C_35_H_52_Cl_2_O_6_ ([M − H]^−^ = 621.3102), clearly lacked one of the methoxy groups found in nostocyclophane D. Based on analyses of ^1^H and ^13^C NMR chemical shifts, C-14 was no longer oxygenated and now corresponded to a methylene. Analyses of the 2D NMR data supported this conclusion. Specifically, the HSQC spectrum revealed an additional aliphatic methylene carbon at 31.2 ppm that correlated to a proton multiplet at 2.40 ppm. This proton signal, in turn, showed HMBC correlations to three sp^2^ carbons (C-10, -11, and -12), establishing the benzylic location of the modification. The types of modifications observed for **2** and **3** have been previously observed [4].

**Figure 2:**
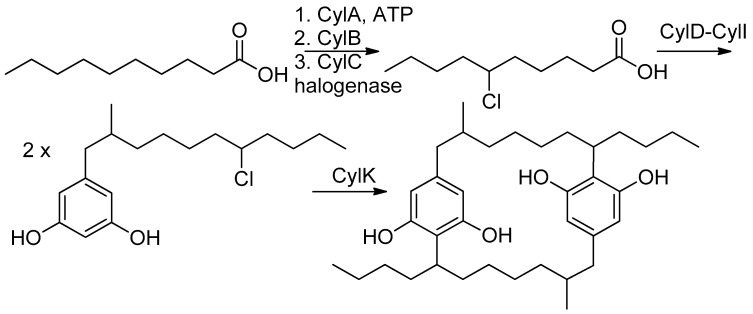
Established biosynthesis for cylindrocyclophane F [16].

Comparison of the NMR spectra and MS spectrometric data for nostocyclophane H (**4**) (Table 1 and Table 2) with the other nostocyclophane analogs isolated revealed an unusual modification in this compound. The molecular formula of **4** was C_34_H_50_Cl_2_O_6_ based on the ion observed at [M − H]^−^ = 623.2897 Da. This formula indicated **4** was smaller than nostocyclophane D by C_2_H_4_, which, given the previously observed modifications, was initially attributed to the lack of two O-methylations. Further analysis of the ^1^H NMR spectrum indicated this assumption was incorrect, as signals for both methoxy groups were observed. Analysis of the ^13^C NMR spectrum also indicated two methylene carbons were missing compared to that of **1**. Based on analysis of the ^1^H-^1^H COSY and HSQC spectra of **4**, it was clear the exocyclic butyl group appended to C-20 in **1** had been truncated to an ethyl group as evidenced by COSY correlations between H_2_-31 to H-20 and H_3_-32. Specifically, methylene carbons at 32.2 and 22.2 ppm were absent from the HSQC spectra of **4**, and one of the triplet methyl signals (H_3_-32) now showed an HMBC correlation to a methine at 37.0 ppm (C-20). To our knowledge, such a modification has not been observed before in the cyanobacterial [7.7]paracyclophanes. Given that these carbons originate from a fatty acid starter unit in the related cylindrocyclophanes, an octanoic starter unit rather than a decanoic acid was likely loaded for one of the monomers (Figure 2). In the case of the cylindrocyclophanes, the corresponding enzyme will accept fatty acids of variable lengths, although it has a clear preference for C-10 fatty acids. In LC-MS competition assays, the loaded enzyme contained a small amount of C-8 (<10%) when the enzyme was presented with equal amounts of C-6 through C-14 saturated fatty acids [9]. The fact that **4** was in relatively low abundance (1.5 mg) compared to the major metabolite nostocyclophane D (200 mg) is consistent with this observation.

Nostocyclophane I (**5**) was obtained as a colorless powder with a molecular formula of C_41_H_62_Cl_2_O_10_ ([M − H]^−^ = 783.3625 Da). The spectroscopic data for **5** were very similar to those of nostocyclophane B. Analysis of the ^1^H and ^13^C NMR spectra (Table 3) indicated that nostocyclophane I existed in two conformations in solution. The observation of two dominant conformers in the NMR spectra of glycosylated nostocyclophane analogs has been previously noted and provided an initial clue as to the identity of **5** [4]. Further analysis of the NMR spectra led to the conclusion that **5** possessed the typical [7.7]paracyclophane core. For example, two benzylic carbons at ~80 ppm could be easily identified by HMBC correlations from the attached methoxy groups. Those oxygenated benzylic methines showed COSY correlations to a methylene at ~4.05 ppm, which then showed COSY correlations to a methylene signal at 2.08 ppm, which further correlated to a proton signal at ~2.8 ppm. The proton chemical shift of that latter proton was consistent with a halide being attached to the corresponding carbon. The main differences in the spectra of **5** and nostocyclophane B could therefore be explained by the presence of a β-xylopyranoside in **5** rather than a D-β-glucopyranoside. Evidence in support of a xylose unit was the presence of an oxygenated methylene (δ_C_ 65.4), expected for this sugar, which showed an HMBC correlation from the anomeric proton (δ_H_ 4.93, d) and a series of consecutive COSY correlations that established the ring, i.e., from that anomeric proton to H-36, from H-36 to H-37, from H-37 to H-38, and then from H-38 to that same oxygenated methylene (H-39). As to the relative configuration of this unit, the equatorial orientations for the hydroxy groups at C-36, C-37 and C-38 were established by a network of ^3^*J*_H,H_ couplings starting at the anomeric proton (H-35, δ_H_ 4.62 d, (7.0 Hz)) through H-38 (δ_H_ 3.29, ddd (10.9, 8.8, 5.0 Hz)). The β-configuration of the sugar was established by the observation of a 7.0 Hz coupling, consistent with the data reported for hexyl β-xylopyranoside, between the anomeric proton H-35 and H-36 as opposed to the 3.5 Hz coupling observed for the α-configuration [17]. Interestingly, this is the first report of a different sugar unit within the nostocyclophane class of [7.7]paracyclophanes, as nostocyclophanes A-B contain a D-glucose unit.

Another nostocyclophane analog that did not fit the typical structural pattern was isolated from the extract. An initial inspection of the NMR data revealed the compound was asymmetrical, given the number of resonances observed. In addition, the ^1^H NMR spectrum of **6** contained a total of five aromatic resonances rather than four as observed in the other asymmetrical analogs, e.g., nostocyclophane D. HRMS data provide another valuable clue as **6** provided an isotope pattern consistent with the incorporation of three chlorine atoms, one more than previously observed for any of the known nostocyclophanes. Analyses of ^1^H-^1^H COSY, HSQC, and HMBC spectra, which were collected in CDCl_3_ as it provided better dispersion in this case, established that **6** lacked the characteristic linkage between C-20 and C-21 and was, therefore, an uncyclized version of nostocyclophane D. In particular, the HSQC spectrum clearly indicated the presence of a new signal for an aromatic methine at 6.09 ppm (H-21) and a new downfield methine signal at δ_H_ 3.97 (H-20) that showed a ^1^*J*_CH_ to a carbon at 64.5 ppm. The latter aromatic signal showed HMBC correlations to two other aromatic signals (C-22 and C-21), which in turn could be connected to a benzylic carbon (C-1) bearing one of the methoxy groups using HMBC data. In contrast, the signal at 3.94 ppm (H-20) showed COSY correlations only to signals indicative of aliphatic protons.

The relative and absolute configurations of **1**–**6** are assumed to be analogous to those of the other members of this series, also isolated from this strain. The configuration of nostocyclophane D produced by this strain (UTEX B1932) was established by X-ray crystallographic analysis [3], as NOE analysis is expected to provide no useful transannular information due to the distance between each set of isolated stereocenters. It was subsequently shown by ECD analysis that nostocyclophanes A-D exhibited similar ECD curves, indicating that the other analogs had identical configurations to nostocyclophane D [4]. Not surprisingly, the ECD curves for nostocyclophane D and **1**–**5** have identical Cotton effects as well (See Figure S33), with a strong negative Cotton effect at around 214, a shoulder at 230 nm, and two broad weakly negative effects between 260 and 280 nm. The ECD spectrum of the linear analog **6** (see Figure S34) shows similar Cotton effects to **1**–**5**, consistent with the report of the similarity between the ECD spectra of linear and cyclic forms of the cylindrofridins [7]. Based on their shared biosynthesis, **1**–**6** are expected to share the same configurations as nostocyclophane D at the conserved chiral centers. Regarding the final chiral center in **6**, at this time, the configuration of C-20 has not been established. In the cylindrocyclophane biosynthesis, an *R*-configuration is required at this position of the fatty acid unit, and this sp^3^ center undergoes an inversion of configuration during the cyclization process [16]. A similar mechanism likely operates in the nostocyclophane biosynthesis, but it is also possible that the linear analog **6** possesses the “incorrect” configuration at C-20 and is, therefore, unable to cyclize. The corresponding cylindrocyclophane enzyme cannot cyclize fatty acids with an *S*-configuration [16]. Thus, C-20 in **6** remains unassigned, as it is for the other linear halogenated analog cylindrofridin B [7]. Due to the isolated nature of this center, the chemical shift differences between the C-20 diastereomers are likely to be minor [18], so determining that center’s configuration would require synthesis or X-ray crystallography rather than computational approaches relying on NMR spectroscopy.

Known cyanobacterial [7.7]paracyclophanes are divided into three basic structural families. The cylindrocyclophanes generally have hydroxylation and methylation at C-1 and C-2, respectively. In contrast, the core of the merocyclophanes is only decorated with methyl groups at C-1/C-14. Finally, the nostocyclophanes have hydroxy and chloro groups at C-1/C-14 and C-3/C-16, respectively. Linear analogs within the cylindrocyclophane family have been previously reported by Preisitsch et al. [7]. In that case, cylindrofridin A is the monomeric halogenated alkyl resorcinol, i.e., the monomer, while B and C result from a single coupling between the aromatic ring and the alkyl chain similar to nostocyclophane J, i.e., dimeric but uncyclized. The linear cylindrofridins were the first examples of this likely biosynthetic intermediate within the [7.7.]paracyclophanes structural family.

Dedichloronostocyclophane D, which notably lacked any chlorine atoms, has been previously reported from the sea slug, *Planaxis sulcatus* [14] and had not been reported from any cyanobacterial strain before. That report represents the first time that naturally occurring [7.7]paracyclophanes have been reported from a sea slug. Its presence is likely due to the slug feasting on cyanobacteria, as observed for other compounds [19,20].

Nostocyclophane C and **1**–**6** displayed moderate to weak growth inhibition against breast epithelial adenocarcinoma MDA-MB-231 cells (Table 4). The observed activity and level of potency are generally consistent with previous observations for naturally occurring [7.7]paracyclophanes. For example, nostocyclophanes A–C were cytotoxic at 1–2 μg/mL, while nostocyclophane D was slightly more potent (GI_50_ = ~0.76 μM) against KB (human nasopharyngeal carcinoma) and LoVo (human colon adenocarcinoma) cells [4], similar to the cylindrocyclophanes A–F (0.5–5 μg/mL). Related compounds, the merocyclophanes, were inhibitory to HT-29, NCI-H460, SF268, and MCF7 cancer cells with GI_50_ values of ~1–10 μM.

In conclusion, the structures of six new nostocyclophanes were determined by NMR and MS techniques. Of particular interest is nostocyclophane J, a linear analog in which one of the “cryptic” halogenations is evident, and nostocyclophane H, derived from two different alkyl resorcinol intermediates. These compounds provide interesting insights into the biosynthesis and substrate tolerance for *Nostoc* sp. B1932 and the way in which it compares to other reported [7.7]paracyclophanes. Compounds **1**–**6** mildly inhibited the growth of MDA-MB-231 cells, a level of activity consistent with what has been reported for other naturally occurring [7.7]paracyclophanes.

## 3. Materials and Methods

### 3.1. General Experimental Procedures

Optical rotations were measured on a Jasco-DIP-700 polarimeter at the sodium line (589 nm). UV spectra were obtained on a Hewlett-Packard 8453 spectrophotometer and IR spectra were measured as a thin film on a CaF_2_ disc using a Perkin Elmer 1600 series FTIR. ECD measurements were obtained on a Chirascan Circular Dichroism Spectrometer with the samples dissolved in MeOH and placed in a 1 cm quartz cuvette with a solvent subtraction for baseline correction. NMR spectra were acquired on a Varian Inova Unity 500 MHz spectrometer operating at 500 (^1^H) or 125 (^13^C) MHz using the residual solvent signals as an internal reference. Samples were in 3 mm Shigemi tubes during NMR analyses. High-resolution mass spectrometry data were obtained on an Agilent LC-TOF or LC-QTOF with ES ionization. Gradient separations used a Shimadzu system consisting of LC-20AT Solvent Delivery Modules, an SPD-M20A VP Diode Photodiode Array Detector, and a SCL-20A VP System Controller. TLC analyses were performed on Si_60_F_254_ plates and visualized under UV or by heating after spraying with a 1% anisaldehyde solution in acetic acid:H_2_SO_4_ (50:1). Samples were weighted on a Mettler Toledo analytical balance.

### 3.2. Chemicals and Reagents

ACS grade solvents (Fisher Scientific, Waltham, MA, USA) were distilled and filtered before use. HP-20 resin, flash column stationary phases, and HPLC columns were purchased from Supelco (St. Louis, MO, USA), Sorbent Technologies (Atlanta, GA, USA), and Phenomenex (Torrance, CA, USA), respectively.

### 3.3. Biological Material

Cyanobacterial sample UTEX B1932, *Nostoc linckia*, was originally purchased from the University of Texas Culture Collection. UTEX identified the strain before our purchase. After recovery from cryopreservation, the bacterium was cultured at a temperature of 24 ± 1 °C in 20 L glass carboys containing A_3_M_7_ media [21]. Cultures were continuously illuminated at an incident intensity of 100–200 µmol·m^−2^·s^−1^ from banks of fluorescent tubes and aerated at 0.20–0.25 times the culture volume per minute (4–5 L/min) with 0.5% CO_2_ in air. After 37 days, the cyanobacterial cultures were harvested through 210 µm plastic mesh by recirculating the culture via a peristaltic pump until the filtrate was clear. The retained cyanobacterial cell mass was then freeze-dried, yielding between 0.11 and 0.36 g/L of biomass.

### 3.4. Extraction and Isolation of Metabolites

Freeze-dried alga (35 g) was extracted three times with 2 L of 7:3 EtOH:H_2_O for 24 h at room temperature. The combined dark green extract (10 g) was concentrated in vacuo to 350 mL and the concentrate applied to a 25 × 4.6 cm column of ODS silica gel. The column was eluted with 1:1 MeOH:H_2_O (1.5 L), 4:1 MeOH:H_2_O (2 L), and MeOH (2 L). The 4:1 MeOH:H_2_O fraction was evaporated. The residue (1.3 g) was further purified by RP-HPLC (Luna C18, 250 × 10 mm, a linear gradient from 50 to 100% MeCN in water over 40 min and then 100% MeCN for 20 min) to afford 40 mg of nostocyclophane B (0.4% yield, 95.6% purity, *t*_R_ 8.1 min), 5.0 mg of nostocyclophane I (0.05% yield, 98.6% purity, *t*_R_ 10.1 min), 2.0 mg of nostocyclophane F (0.02% yield, 93.7% purity, *t*_R_ 12.5 min), 6 mg of nostocyclophane C (0.06% yield, 97.6% purity, *t*_R_ 14.2 min), 1.0 mg of nostocyclophane H (0.01% yield, 97.6% purity, *t*_R_ 16.0 min), 3 mg of dedichloronostocyclophane D (0.03% yield, 98.9% purity, *t*_R_ 17.2 min), 20 mg of nostocyclophane E (0.2% yield, 99.2% purity, *t*_R_ 19.5 min), 200 mg of nostocyclophane D (2% yield, 95.2% purity, *t*_R_ 22.5 min), 1.0 mg of nostocyclophane G (0.01% yield, 95.2% purity, *t*_R_ 26.5 min), and 1.5 mg of nostocyclophane J (0.015% yield, 95.7% purity, *t*_R_ 27.5 min).

Nostocyclophane E (**1**): amorphous white power; [α]_D_^22^ +5.7 (*c* 2.0, MeOH); UV (MeOH) λ_max_ (log ε) 209 (2.5), 214 (2.5), 227 (1.3), 277 (0.2), 283 (0.2) nm; ECD (*c* 0.2 MeOH) λ_max_ (Δ ε) 206 (0.80), 218 (−0.71), 240 (−0.20), 261 (−0.80), 284 (−0.090) nm; IR (film) *ν*_max_ 3325, 2926, 2854, 1646, 1595, 1431, 1083, 1021 cm^−1^; see Table 1 and Table 2 for tabulated NMR spectroscopic data; HRESIMS *m*/*z* 617.3599 [M − H]^−^ (calcd for C_36_H_54_^35^ClO_6_, 617.3609; Δ = −1.6 ppm).
Nostocyclophane F (**2**): amorphous white power; [α]_D_^22^ −6.5 (*c* 0.2, MeOH); UV (MeOH) λ_max_ (log ε) 210 (2.5), 214 (2.5), 227 (1.4), 277 (0.3), 283 (0.2) nm; ECD (*c* 0.2 MeOH) λ_max_ (Δ ε) 205 (1.10), 217 (−1.80), 235 (−0.67), 272 (−0.26), 280 (−0.25) nm; IR (film) *ν*_max_ 3345, 2958, 2864, 1650, 1431, 1020 cm^−1^; see Table 1 and Table 2 for tabulated NMR spectroscopic data; HRESIMS *m*/*z* 603.3454 [M − H]^−^ (calcd for C_35_H_52_^35^ClO_6_^−^, 603.3453; Δ = 0.3 ppm).
Nostocyclophane G (**3**): amorphous white power; [α]_D_^22^ −25.6 (*c* 2.0, MeOH); UV (MeOH) λ_max_ (log ε) 208 (0.9), 229 (0.3), 274 (0.1) nm; ECD (*c* 0.2 MeOH) λ_max_ (Δ ε) 202 (0.9), 217 (−0.20), 237 (−0.89), 285 (−0.02) nm; IR (film) *ν*_max_ 3390, 2932, 2859, 1588, 1429, 1085 cm^−1^; see Table 1 and Table 2 for tabulated NMR spectroscopic data; HRESIMS *m*/*z* 621.3102 [M − H]^−^ (calcd for C_35_H_51_^35^Cl_2_O_5_^−^, 621.3114; Δ = −1.9 ppm).
Nostocyclophane H (**4**): amorphous white power; [α]_D_^22^ −0.2 (*c* 2.0, MeOH); UV (MeOH) λ_max_ (log ε) 209 (2.4), 227 (0.9), 274 (0.2), 283 (0.2) nm; ECD (*c* 0.2 MeOH) λ_max_ (Δ ε) 207 (0.22), 218 (−0.25), 233 (−0.09), 273 (−0.35), 282 (−0.37) nm; IR (film) *ν*_max_ 3399, 2932, 2873, 1589, 1428, 1089 cm^−1^; see Table 1 and Table 2 for tabulated NMR spectroscopic data; HRESIMS *m*/*z* 623.2901 [M − H]^−^ (calcd for C_34_H_49_^35^Cl_2_O_6_^−^, 623.2906; Δ = −0.8 ppm).
Nostocyclophane I (**5**): amorphous white power; [α]_D_^22^ +2.0 (*c* 1.0, MeOH); UV (MeOH) λ_max_ (log ε) 209 (2.0), 227 (0.6), 275 (0.1), 283 (0.1) nm; ECD (*c* 0.2 MeOH) λ_max_ (Δ ε) 201 (4.9), 215 (−5.0), 233 (−1.45), 271 (−0.35), 283 (−0.34) nm; IR (film) *ν*_max_ 3341, 2927, 2863, 1614, 1595, 1429, 1093 cm^−1^; see Table 3 for tabulated NMR spectroscopic data; HRESIMS *m*/*z* 783.3642 [M − H]^−^ (calcd for C_41_H_61_^35^Cl_2_O_10_^−^, 783.3642; Δ = −2.1 ppm).
Nostocyclophane J (**6**): amorphous white power; [α]_D_^22^ +5.0 (*c* 1.0, MeOH); UV (MeOH) λ_max_ (log ε) 214 (2.6), 228 (1.7), 278 (0.4), 282 (0.4) nm; ECD (*c* 0.2 MeOH) λ_max_ (Δ ε) 207 (2.6), 217 (−2.2), 228 (−0.8), 273 (−0.44), 293 (−0.37) nm. IR (film) *ν*_max_ 3374, 2950, 2864, 1604, 1081 cm^−1^; see Table 5 for tabulated NMR spectroscopic data; HRESIMS *m*/*z* 687.2973 [M − H]^−^ (calcd for C_36_H_54_^35^Cl_3_O_6_^−^, 687.2986; Δ = −1.9 ppm).

### 3.5. Growth Inhibition Assays

MDA-MB-231 cell lines were maintained in DMEM media supplemented with 10% premium fetal bovine serum and 50 U/mL penicillin and 50 μg/mL streptomycin. One day before treatment, cancer cells were seeded at 2000 cells per well into a 96-well tissue culture plate. Twenty four hours post-seeding, the serially diluted compounds and vehicle (DMSO) controls were added to the cells for the cytotoxicity assay, and incubated at 37 °C with 5% CO_2_ for 72 h. Then, equal volume of MTT dye (Alfa Aesar, Teksbury, MA, USA) was added to each well and incubated with 5% CO_2_ at 37 °C for 60 min. Next, media were aspirated and 100 μL of DMSO were added to each well to dissolve formazan crystals. Cell viability data were collected with a Victor Nivo Multimode Microplate Reader (Perkin Elmer, Waltham, MA, USA) at 570 nm and GI_50_ curves were generated using GraphPad Prism 5. For GI_50_ determination, samples were tested in triplicate on the same plate at each concentration, and the results averaged. Three consecutive biological replicates were collected and average GI_50_ values were calculated. Tamoxifen (1 μM final) and Gemcitabine (1 μM final) were used as positive controls for each replicate. The MDA-MB 231 cells were obtained from the University of Hawaii Cancer Center which they obtained as part of the NCI-60 set of cell lines from National Cancer Insti-tute.

## Figures and Tables

**Table 1:** ^1^H NMR Spectroscopic Data (500 MHz, δ_H_ (*J* in Hz)) for compounds **1**–**4** (DMSO-*d*_6_).

Position	1	2	3	4
	δ_H_ (*J* in Hz)	δ_H_ (*J* in Hz)	δ_H_ (*J* in Hz)	δ_H_ (*J* in Hz)
1	4.04, dd (10.7, 3.9)	4.03, dd (10.5, 3.9)	4.04, dd (10.7, 4.1)	4.03, dd (10.8, 3.9)
2	2.07, ddd (11.0, 10.7, 3.9)	2.07, ddd (11.1, 10.1, 3.8)	2.08, ddd (11.2, 10.2, 3.3)	2.07, ddd (13.2, 10.8, 3.9)
	1.82, m	1.83, m	1.82, m	1.83, m
3	2.82, t (10.7)	2.82, t (10.1)	2.83, t (10.2)	2.80, t (10.8)
4	1.59, m	1.59, m	1.59, m	1.61, m
	1.46, m	1.45, m	1.46, m	1.46, m
5	1.36, m	1.36, m	1.36, m	1.35, m
	0.52, m	0.53, m	0.58, m	0.54, m
6	1.82, m	1.82, m	1.82, m	1.83, m
	1.52, m	1.50, m	1.55, m	1.52, m
7	3.07, m	3.07, m	3.09, m	3.07, m
10	6.09, s	6.09, s	6.06, s	6.10, s
12	6.13, s	6.19, s	6.15, s	6.15, s
14	3.69, dd (10.3, 4.4)	4.05, dd (9.7, 4.0)	2.40, m	4.03, dd (10.8, 3.9)
15	1.66, m	1.60, m	1.60, m	2.07, ddd (13.2, 10.8, 3.9)
	1.30, m	1.30, m		1.83, m
16	1.11, m	1.11, m	2.54, m	2.80, t (10.8)
	1.00, m	1.00, m		
17	0.98, m	0.98, m	1.59, m	1.61, m
	0.51, m	0.52, m	1.46, m	1.46, m
18	0.88, m	0.89, m	1.36, m	1.35, m
	0.32, m	0.32, m	0.58, m	0.54, m
19	1.82, m	1.82, m	1.82, m	1.83, m
	1.52, m	1.52, m	1.55, m	1.52, m
20	3.07, m	3.07, m	3.09, m	3.07, m
23	6.08, s	6.13, s	6.10, s	6.10, s
25	5.99, s	5.95, s	5.93, s	6.15, s
27	1.80, m	1.80, m	1.82, m	1.80, m
	1.38, m	1.30, m	1.38, m	1.35, m
28	1.18, m	1.18, m	1.18, m	1.18, m
29	1.25, m	1.25, m	1.25, m	1.23, m
30	0.77, t (7.2)	0.76, t (7.2)	0.77, t (7.2)	0.76, t (7.2)
31	1.80, m	1.80, m	1.82, m	1.80, m
	1.38, m	1.30, m	1.38, m	1.54, m
32	1.18, m	1.18, m	1.18, m	0.69, t (7.4)
33	1.25, m	1.25, m	1.25, m	
34	0.77, t (7.2)	0.76, t (7.2)	0.77, t (7.2)	
1-OMe	3.03, s	3.03, s	3.03, s	3.07, s
14-OMe	3.01, s			3.07, s

**Table 2:** ^13^C NMR Spectroscopic Data (125 MHz) for **1**–**4** (DMSO-*d*_6_).

Position	1	2	3	4
	δ_C_, Mult.	δ_C_, Mult.	δ_C_, Mult.	δ_C_, Mult.
1	81.0, CH	81.0, CH	81.0, CH	80.9, CH
2	45.5, CH_2_	45.5, CH_2_	45.5, CH_2_	45.5, CH_2_
3	62.9, CH	62.9, CH	62.9, CH	62.8, CH
4	40.3, CH_2_	40.0, CH_2_	40.0, CH_2_	40.2, CH_2_
5	26.8, CH_2_	26.6, CH_2_	26.7, CH_2_	26.7, CH_2_
6	33.3, CH_2_	33.4, CH_2_	33.2, CH_2_	32.7, CH_2_
7	34.8, CH	34.9, CH	34.7, CH	34.9, CH
8	115.5, qC	114.7, qC	116.2, qC	115.8, qC
9	155.6, qC	155.4, qC	155.7, qC	155.7, qC
10	102.8, CH	103.5, CH	102.8, CH	102.8, CH
11	139.6, qC	143.6, qC	137.7, qC	138.1, qC
12	107.5, CH	107.3, CH	107.3, CH	107.3, CH
13	157.6, qC	157.1, qC	157.7, qC	157.5, qC
14	83.6, CH	73.5, CH	31.2, CH_2_	80.9, CH
15	37.0, CH_2_	39.0, CH_2_	40.0, CH_2_	45.5, CH_2_
16	30.1, CH_2_	30.2, CH_2_	64.2, CH	62.8, CH
17	30.2, CH_2_	30.7, CH_2_	40.0, CH_2_	40.2, CH_2_
18	26.6, CH_2_	27.0, CH_2_	27.0, CH_2_	26.7, CH_2_
19	32.7, CH_2_	32.8, CH_2_	33.2, CH_2_	32.5, CH_2_
20	34.9, CH	34.9, CH	34.8, CH	37.0, CH
21	116.3, qC	116.4, qC	116.2, qC	115.8, qC
22	155.5, qC	155.7, qC	155.7, qC	155.7, qC
23	102.8, CH	102.8, CH	102.8, CH	102.8, CH
24	137.9, qC	138.0, qC	137.7, qC	138.1, qC
25	107.2, CH	106.1, CH	107.3, CH	107.3, CH
26	157.3, qC	157.6, qC	157.7, qC	157.5, qC
27	32.7, CH_2_	33.0, CH_2_	32.8, CH_2_	32.8, CH_2_
28	29.5, CH_2_	29.6, CH_2_	30.2, CH_2_	30.2, CH_2_
29	22.3, CH_2_	22.2, CH_2_	22.2, CH_2_	22.2, CH_2_
30	14.1, CH_3_	14.1, CH_3_	14.1, CH_3_	14.0, CH_3_
31	32.9, CH_2_	33.3, CH_2_	32.8, CH_2_	25.8, CH_2_
32	30.5, CH_2_	30.2, CH_2_	30.2, CH_2_	13.0, CH_3_
33	22.2, CH_2_	22.3, CH_2_	22.2, CH_2_	
34	14.1, CH_3_	14.1, CH_3_	14.1, CH_3_	
1-OMe	55.7, CH_3_	55.7, CH_3_	55.8, CH_3_	55.7, CH_3_
14-OMe	55.5, CH_3_			

**Table 3:** ^1^H and ^13^C NMR Spectroscopic Data for **5** (DMSO-*d*_6_).

Position	5, Major	5, Major	5, Minor	5, Minor
	δ_H_ (*J* in Hz)	δc, Mult.	δ_H_ (*J* in Hz)	δc, Mult.
1	4.05, dd (10.7, 3.4)	81.0, CH	4.04, dd (10.7, 3.9)	80.9, CH
2	2.08, m	45.6, CH_2_	2.06, m	45.5, CH_2_
	1.68, m		1.80, m	
3	2.79, m	62.7, CH	2.82, m	62.6, CH
4	1.58, m	39.6, CH_2_	1.47, m	39.6, CH_2_
	1.42, m		1.36, m	
5	1.46, m	26.7, CH_2_	1.34, m	27.0, CH_2_
	0.53, m		0.45, m	
6	1.84, m	32.4, CH_2_	2.12, m	32.4, CH_2_
	1.55, m		1.58, m	
7	3.10, m	35.1, CH	3.08, m	34.9, CH
8		119.7, qC		118.2, qC
9		157.8, qC		157.5, qC
10	6.32, s	100.8, CH	6.42, s	102.6, CH
11		138.4, qC		138.5, qC
12	6.31, s	107.4, CH	6.30, s	109.3, CH
13		155.8, qC		156.2, qC
14	4.10, dd (10.7, 3.8)	81.2, CH	4.15, dd (10.7, 3.9)	81.0, CH
15	2.08, m	45.9, CH_2_	2.08, m	45.6, CH_2_
	1.85, m		1.80, m	
16	2.91, m	63.1, CH	2.79, m	62.9, CH
17	1.57, m	40.2, CH_2_	1.47, m	40.2, CH_2_
	1.42, m		1.36, m	
18	1.23, m	26.2, CH_2_	1.27, m	26.8, CH_2_
	0.51, m		0.54, m	
19	1.84, m	33.1, CH_2_	1.85, m	33.0, CH_2_
	1.55, m		1.50, m	
20	3.11, m	35.0, CH	3.11, m	34.8, CH
21		116.2, qC		116.2, qC
22		155.6, qC		155.2, qC
23	6.10, s	102.7, CH	6.09, s	102.9, CH
24		138.2, qC		138.3, qC
25	6.15, s	107.0, CH	6.14, s	107.5, CH
26		157.6, qC		157.3, qC
27	2.09, m	32.7, CH_2_	1.72, m	32.8, CH_2_
	1.50, m		1.35, m	
28	1.18, m	30.2, CH_2_	1.18, m	30.0, CH_2_
	1.02, m		1.02, m	
29	1.22, m	22.3, CH_2_	1.22, m	22.3, CH_2_
	1.13, m		1.13, m	
30	0.77, t (6.5)	14.1, CH_3_	0.77, t (6.5)	14.1, CH_3_
31	1.82, m	32.5, CH_2_	1.72, m	32.6, CH_2_
	1.46, m		1.35, m	
32	1.18, m	30.2, CH_2_	1.18, m	30.2, CH_2_
	1.02, m		1.02, m	
33	1.22, m	22.3, CH_2_	1.22, m	22.6, CH_2_
	1.13, m		1.12, m	
34	0.76, t (6.8)	14.1, CH_3_	0.76, t (6.8)	14.1, CH_3_
35	4.93, d (7.0)	99.6, CH	4.62, d (7.0)	105.6, CH
36	3.19, dd (8.8, 7.0)	73.3, CH	3.19, dd (8.8, 7.0)	73.5, CH
37	3.23, t (8.8)	76.6, CH	3.23, t (8.8)	76.8, CH
38	3.29, ddd (10.9, 8.8, 5.0)	69.3, CH	3.29, ddd (10.9, 8.8, 5.0)	69.6, CH
39	3.67, dd (10.9, 5.0)	65.4, CH_2_	3.58, dd (10.9, 5.0)	65.7, CH_2_
	3.12, t (10.9)		3.12, t (10.9)	
1-OMe	3.04, s	55.9, CH_3_	3.04, s	55.9, CH_3_
14-OMe	3.04, s	55.8, CH_3_	3.04, s	55.8, CH_3_

**Table 4:** Assay Data against MDA-MB-231.

Compound	GI_50_ (μM)
**1**	0.72
**2**	0.94
**3**	5.1
**4**	1.2
**5**	1.7
**6**	8.2
Nostocyclophane C	0.95

**Table 5:** ^1^H and ^13^C NMR Spectroscopic Data for **6**.

Position	6 (DMSO-*d*_6_)		6 (CDCl_3_)	
	δ_H_ (*J* in Hz)	δ_C_, Mult.	δ_H_ (*J* in Hz)	δ_C_, Mult.
1	4.03, m	80.5, CH	4.22, dd (8.7, 5.5)	81.2, CH
2	2.00, m	45.6, CH_2_	2.14, ddd (15.3, 10.5, 5.3)	45.3, CH_2_
	1.83, m		1.87, ddd (15.3, 8.7, 3.6)	
3	3.65, m	61.0, CH	3.52, m	59.89, CH
4	1.68, m	37.5, CH_2_	1.74, m	38.0, CH_2_
5	1.30, m	24.2, CH_2_	1.30, m	24.2, CH_2_
	1.11, m		1.20, m	
6	1.85, m	32.7, CH_2_	1.84, m	33.3, CH_2_
	1.55, m		1.62, m	
7	3.10, m	33.9, CH	3.09, m	35.4, CH
8		116.0, qC		117.5, qC
9		155.5, qC		155.6, qC
10	6.18, s	104.5, CH	6.43, s	106.4, CH
11		138.5, qC		139.1, qC
12	6.09, s	104.5, CH	6.43, s	106.4, CH
13		155.5, qC		155.6, qC
14	4.05, m	80.5, CH	4.27, dd (8.7, 5.3)	81.3, CH
15	2.14, m	45.7, CH_2_	2.28, ddd (14.6, 9.7, 5.5)	44.8, CH_2_
	1.80, m		1.96, ddd (14.6, 8.7, 3.5)	
16	3.35, m	61.1, CH	3.59, m	59.88, CH
17	1.68, m	37.1, CH_2_	1.68, m	37.8, CH_2_
18	1.64, m	23.0, CH_2_	1.72, m	23.2, CH_2_
	1.33, m		1.46, m	
19	1.64, m	36.9, CH_2_	1.60, m	37.9, CH_2_
20	3.97, m	64.5, CH	3.86, m	64.0, CH
21	6.09, s	102.0, CH	6.30, s	102.6, CH
22		158.5, qC		157.0, qC
23	6.11, s	104.5, CH	6.43, s	106.4, CH
24		142.9, qC		143.5, qC
25	6.18, s	104.5, CH	6.43, s	106.4, CH
26		158.5, qC		157.0, qC
27	1.74, m	31.8, CH_2_	1.68, m	38.2, CH_2_
	1.48, m		1.61, m	
28	1.00, m	30.3, CH_2_	1.28, m	30.6, CH_2_
	1.11, m		1.17, m	
29	1.24, m	22.3, CH_2_	1.23, m	22.0, CH_2_
	1.17, m			
30	0.77, t (7.1)	14.1, CH_3_	0.83, t (7.1)	14.1, CH_3_
31	1.64, m	37.0, CH_2_	1.80, m 1.55, m	32.3, CH_2_
32	1.40, m	28.0, CH_2_	1.30, m	28.7, CH_2_
33	1.25, m	21.7, CH_2_	1.35, m 1.30, m	22.2, CH_2_
34	0.85, t (7.2)	13.9, CH_3_	0.90, t (7.2)	13.9, CH_3_
1-OMe	3.04, s	55.8, CH_3_	3.20, s	56.5, CH_3_
14-OMe	3.05, s	55.8, CH_3_	3.26, s	56.6, CH_3_

## Data Availability

The original contributions presented in the study are included in the article/Supplementary Material; further inquiries can be directed to the corresponding author.

## Supplementary Materials

The following supporting information can be downloaded at: https://www.mdpi.com/article/10.3390/md21020101/s1, Figures S1–S34: Copies of the NMR, MS, and ECD spectra for all new compounds.
