# Supplementary material for: New Nostocyclophanes from Nostoc linckia
Source: Mar Drugs. 2023 Jan 31;21(2):101. doi: 10.3390/md21020101 (PMC9967113; doi:10.3390/md21020101)

New Nostocyclophanes from *Nostoc linckia*

TABLE OF CONTENTS

Page

Figure S1. ^{1}H NMR Spectrum (500 MHz) of 1 in $DMSO_6$ 3
Figure S2. ^{13}C NMR Spectrum (125 MHz) of 1 in $DMSO_6$ 4
Figure S3. HSQC NMR Spectrum (500 MHz) of 1 in $DMSO_6$ 5
Figure S4. COSY NMR Spectrum (500 MHz) of 1 in $DMSO_6$ 6
Figure S5. HMBC Spectrum (500 MHz) of 1 in $DMSO_6$ 7
Figure S6. ^{1}H NMR Spectrum (500 MHz) of 2 in $DMSO_6$ 8
Figure S7. ^{13}C NMR Spectrum (125 MHz) of 2 in $DMSO_6$ 9
Figure S8. HSQC NMR Spectrum (500 MHz) of 2 in $DMSO_6$ 10
Figure S9. COSY NMR Spectrum (500 MHz) of 2 in $DMSO_6$ 11
Figure S10. HMBC Spectrum (500 MHz) of 2 in $DMSO_6$ 12
Figure S11. ^{1}H NMR Spectrum (500 MHz) of 3 in $DMSO_6$ 13
Figure S12. ^{13}C NMR Spectrum (125 MHz) of 3 in $DMSO_6$ 14
Figure S13. ^{1}H NMR Spectrum (500 MHz) of 4 in $DMSO_6$ 15
Figure S14. ^{13}C NMR Spectrum (125 MHz) of 4 in $DMSO_6$ 16
Figure S15. HSQC NMR Spectrum (500 MHz) of 4 in $DMSO_6$ 17
Figure S16. COSY NMR Spectrum (500 MHz) of 4 in $DMSO_6$ 18
Figure S17. HMBC Spectrum (500 MHz) of 4 in $DMSO_6$ 19
Figure S18. ^{1}H NMR Spectrum (500 MHz) of 5 in $DMSO_6$ 20
Figure S19. ^{13}C NMR Spectrum (500 MHz) of 5 in $DMSO_6$ 21
Figure S20. ^{1}H NMR Spectrum (500 MHz) of 6 in $CDCl_3$ 22
Figure S21. ^{13}C NMR Spectrum (125 MHz) of 6 in $CDCl_3$ 23
Figure S22. HSQC NMR Spectrum (500 MHz) of 6 in $CDCl_3$ 24
Figure S23. COSY NMR Spectrum (500 MHz) of 6 in $CDCl_3$ 25
Figure S24. HMBC Spectrum (500 MHz) of 6 in $CDCl_3$ 26
Figure S25. ^{1}H NMR Spectrum (500 MHz) of 6 in $DMSO_6$ 27
Figure S26. ^{13}C NMR Spectrum (125 MHz) of 6 in $DMSO_6$ 28
Figure S27. HR-ESI-MS Spectrum of 1 29
Figure S28. HR-ESI-MS Spectrum of 2 30
Figure S29. HR-ESI-MS Spectrum of 3 31
Figure S30. HR-ESI-MS Spectrum of 4 32
Figure S31. HR-ESI-MS Spectrum of 5 33
Figure S32. HR-ESI-MS Spectrum of 6 34
Figure S33. Electronic Circular Dichroism Spectra (MeOH) of Nostocyclophane D, Dedichloronostocyclophane D, and 1-5 35
Figure S34. Electronic Circular Dichroism Spectra (MeOH) of 6 36

Figure S1. ^{1}H NMR Spectrum (500 MHz) of **1** in DMSO$_6$

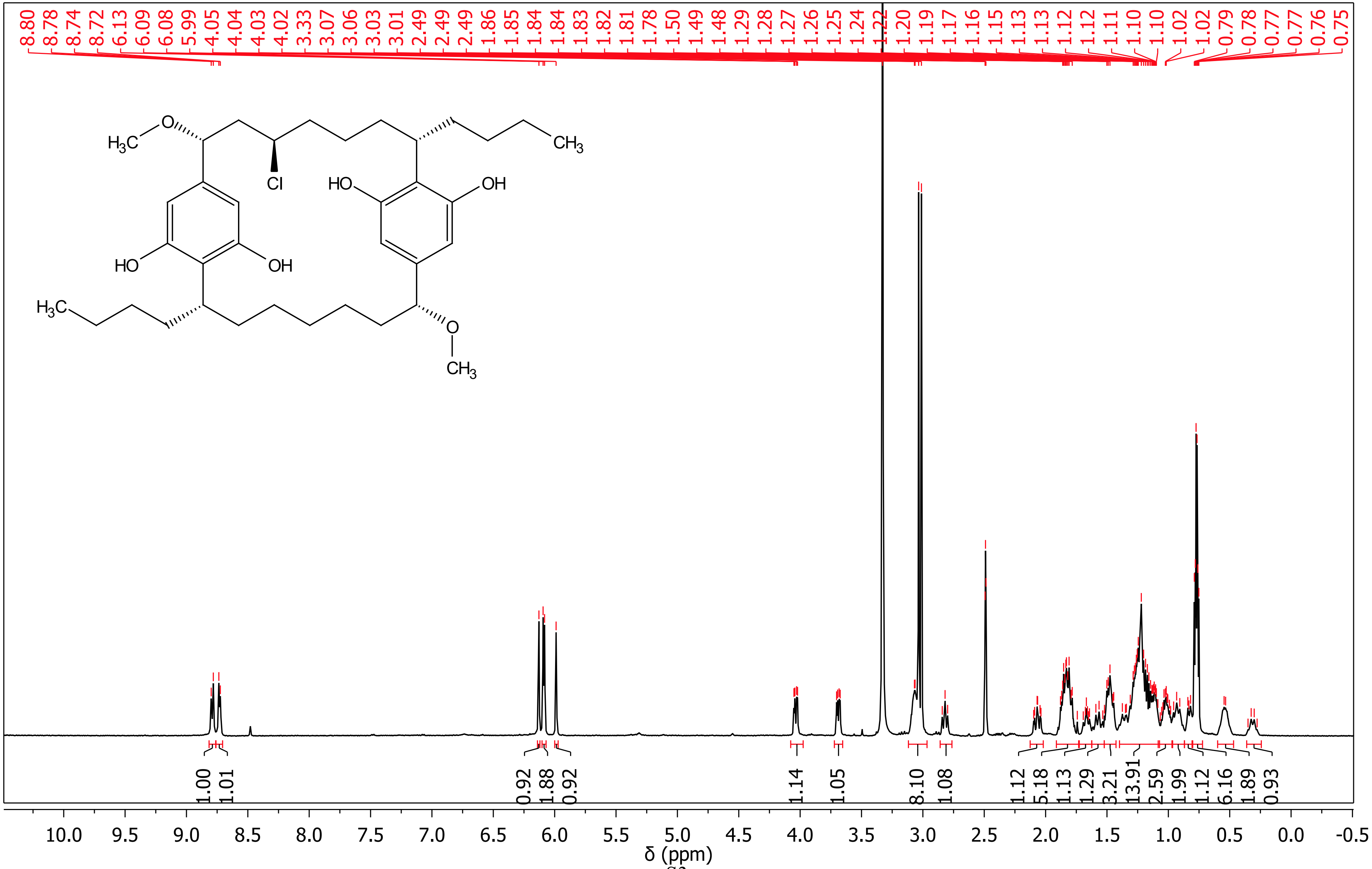

Figure S2. ^{13}C NMR Spectrum (125 MHz) of **1** in DMSO$_6$

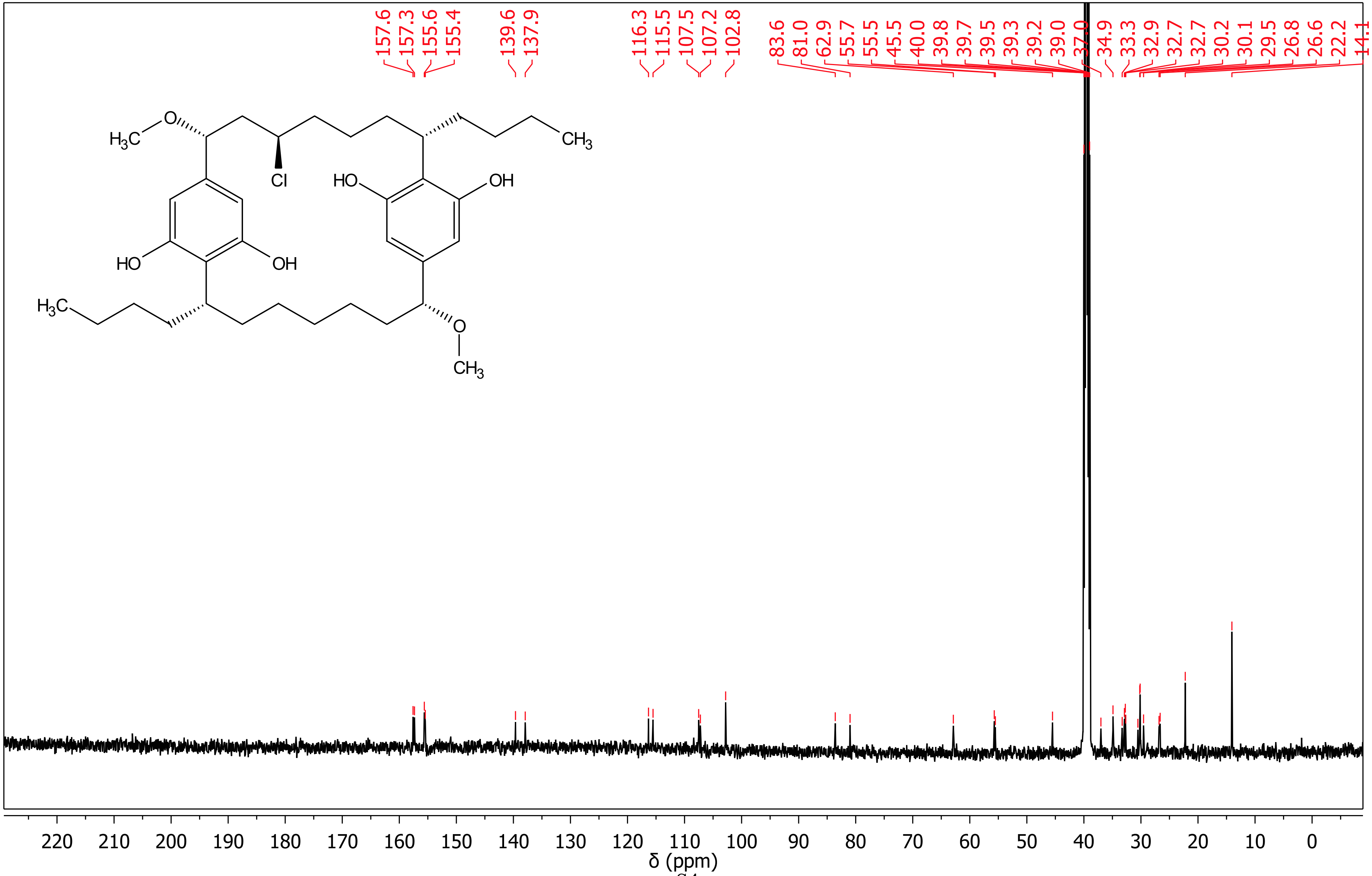

Figure S3. HSQC NMR Spectrum (500 MHz) of **1** in DMSO$_6$

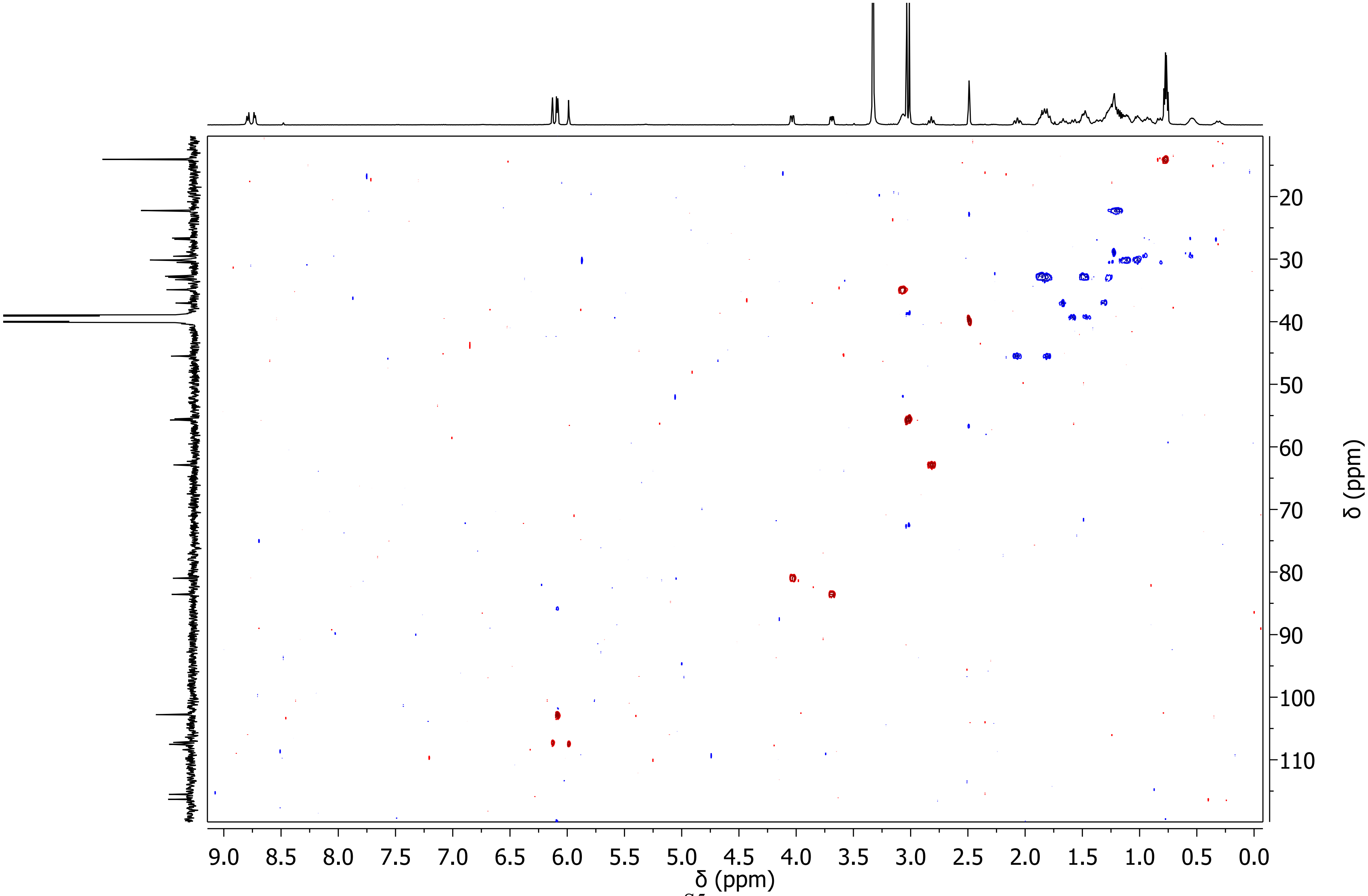

Figure S4. COSY NMR Spectrum (500 MHz) of **1** in DMSO$_6$

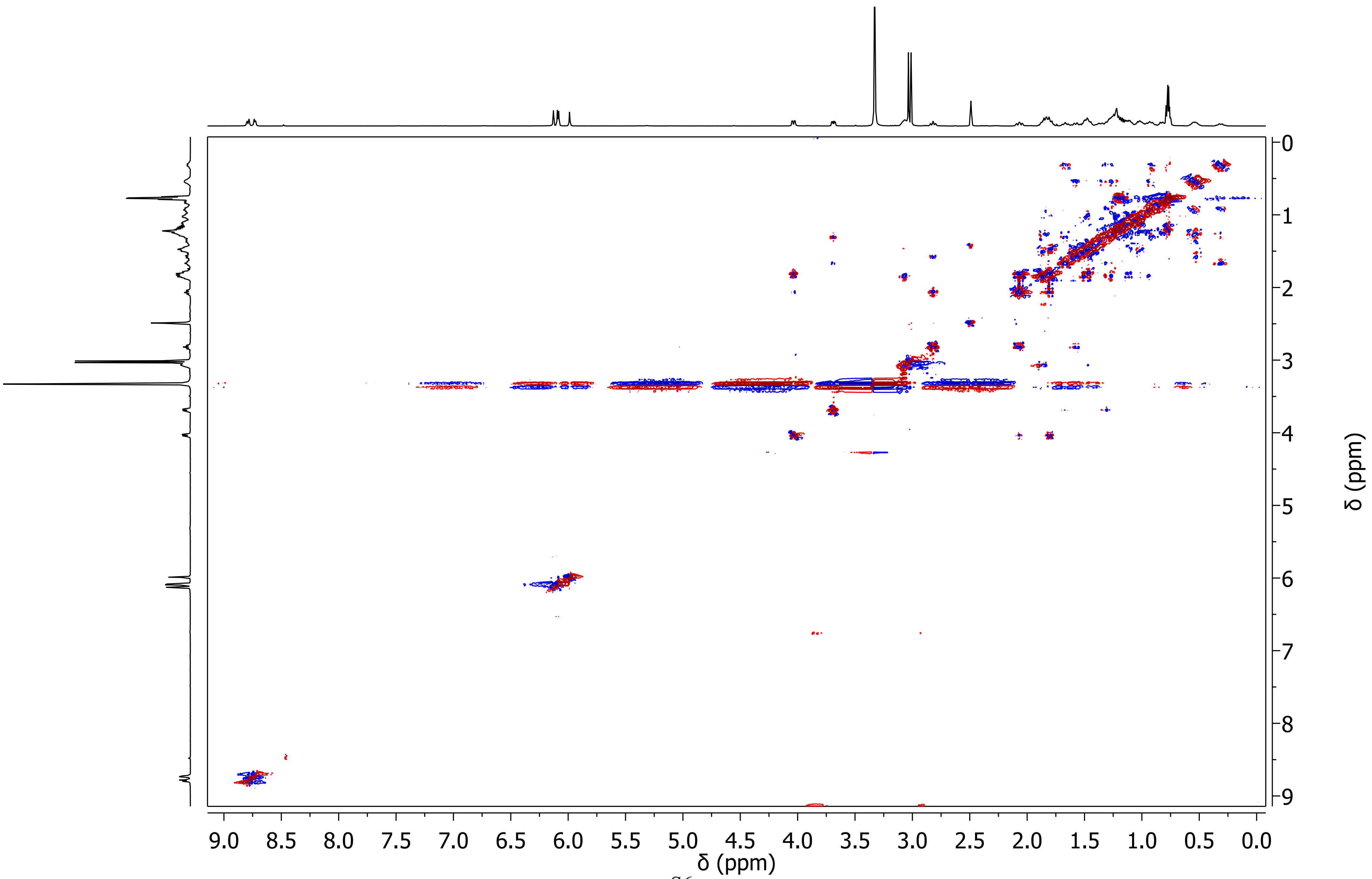

Figure S5. HMBC Spectrum (500 MHz) of **1** in $DMSO_6$

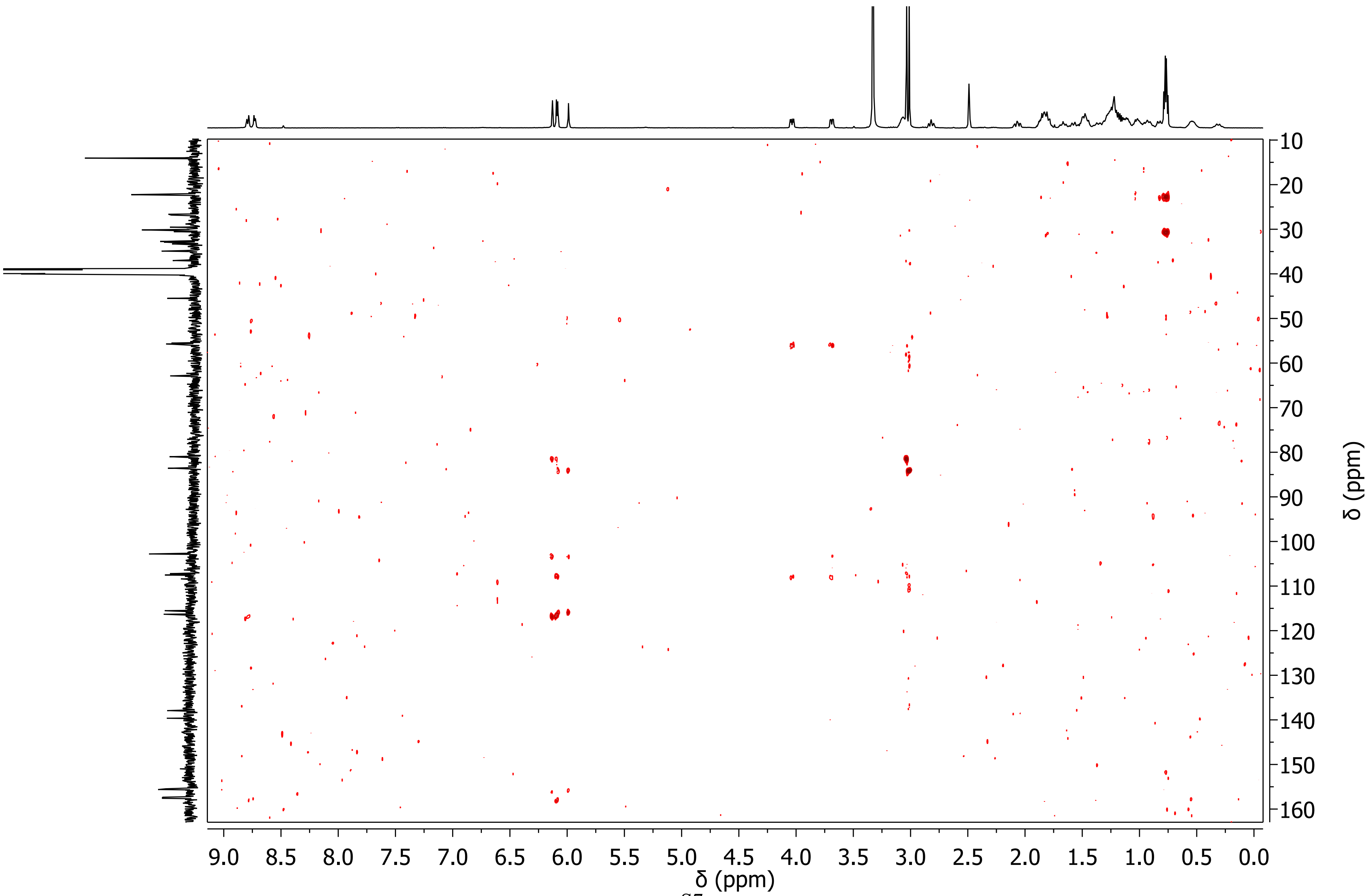

Figure S6. ^{1}H NMR Spectrum (500 MHz) of 2 in DMSO$_6$

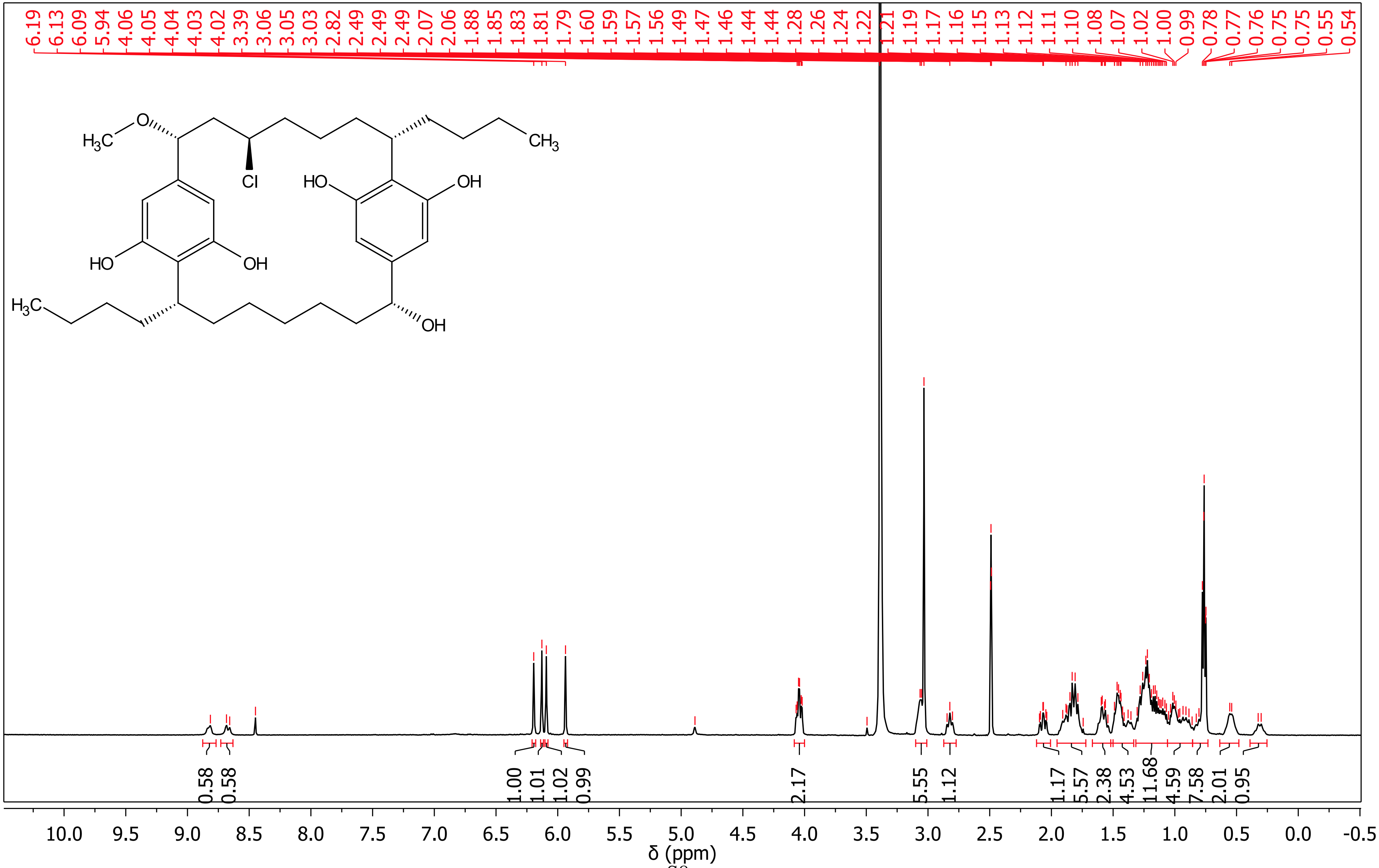

Figure S7. ^{13}C NMR Spectrum (125 MHz) of **2** in DMSO6

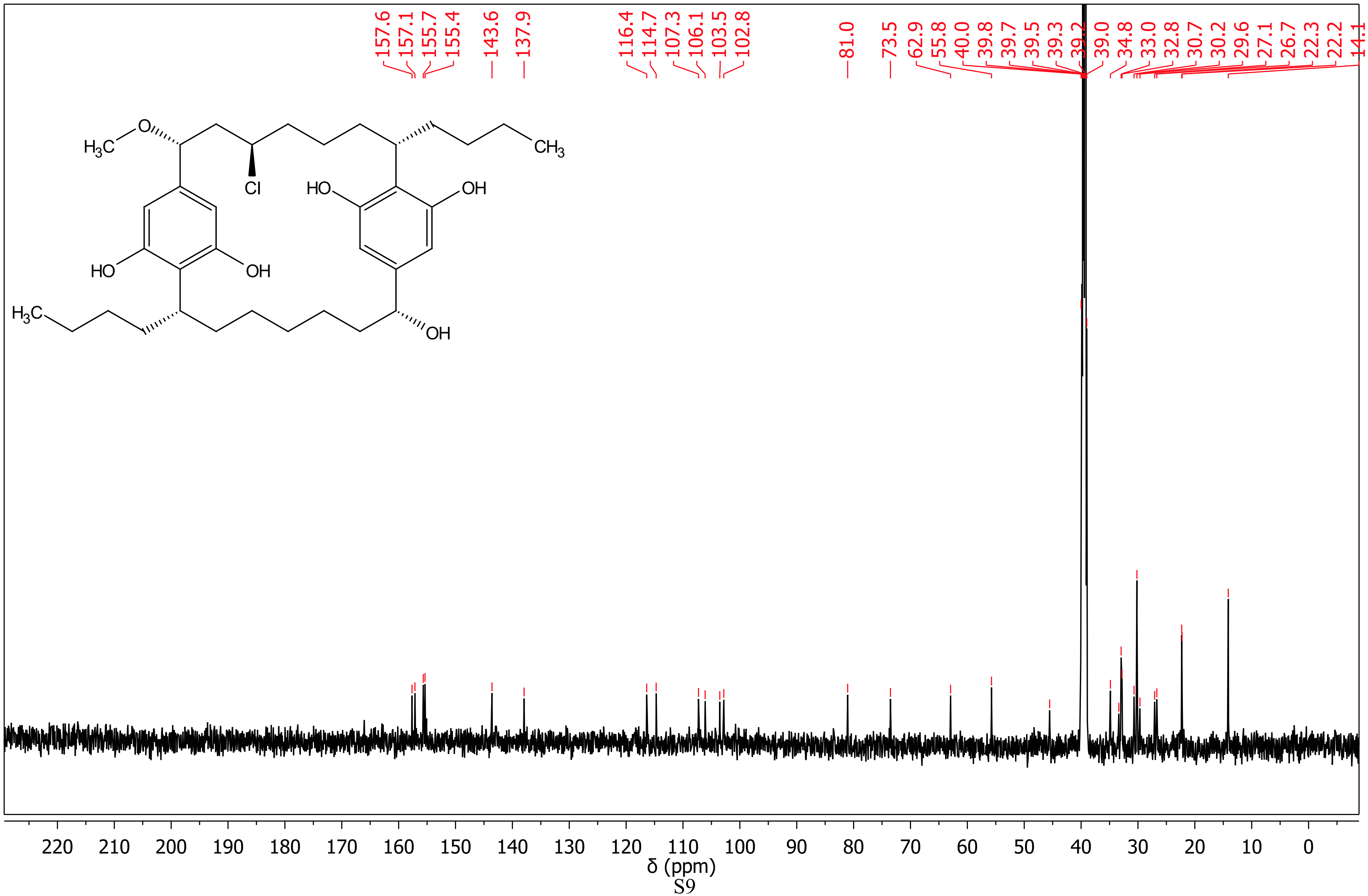

Figure S8. HSQC NMR Spectrum (500 MHz) of **2** in DMSO6

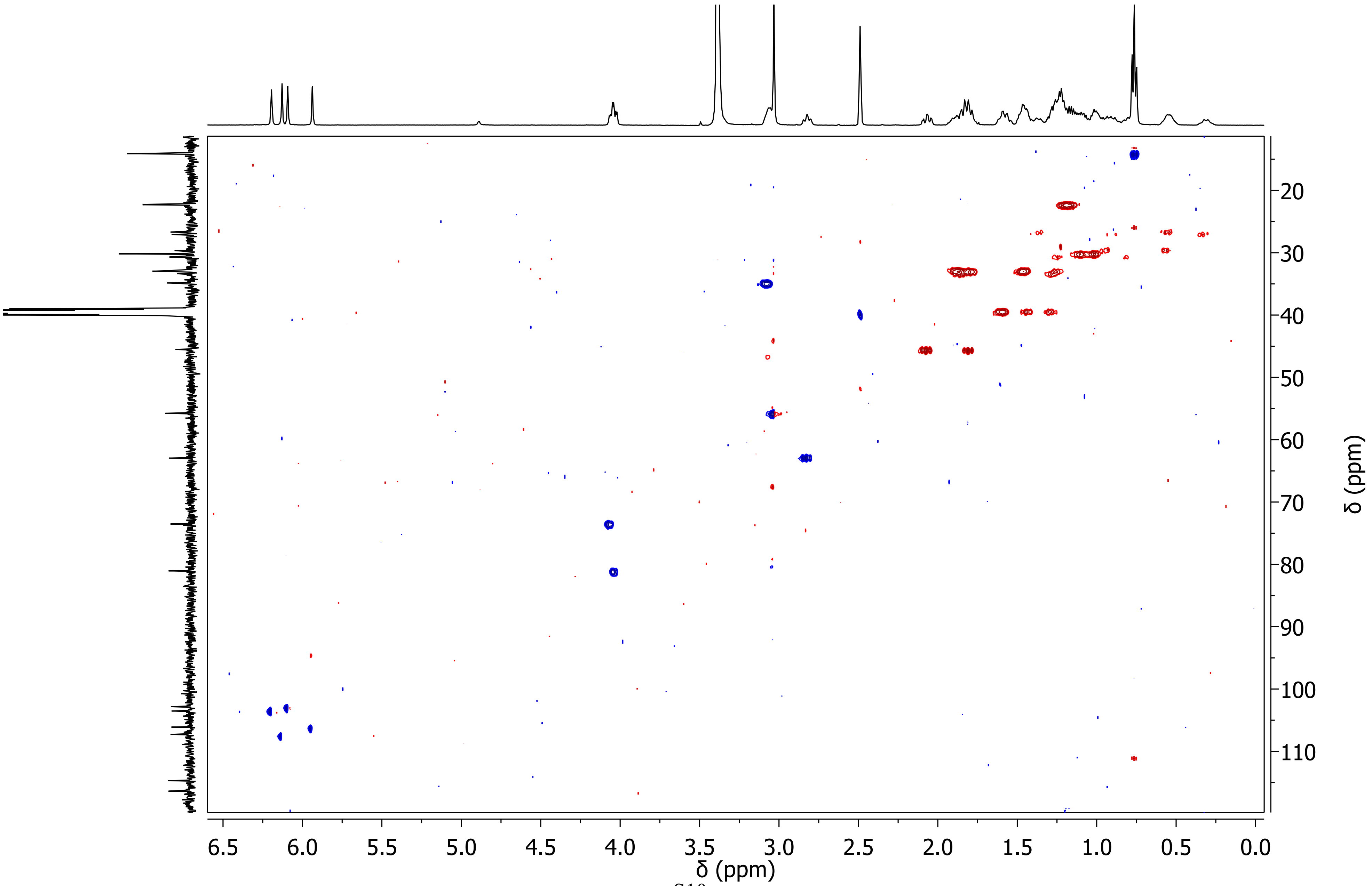

Figure S9. COSY NMR Spectrum (500 MHz) of **2** in DMSO$_6$

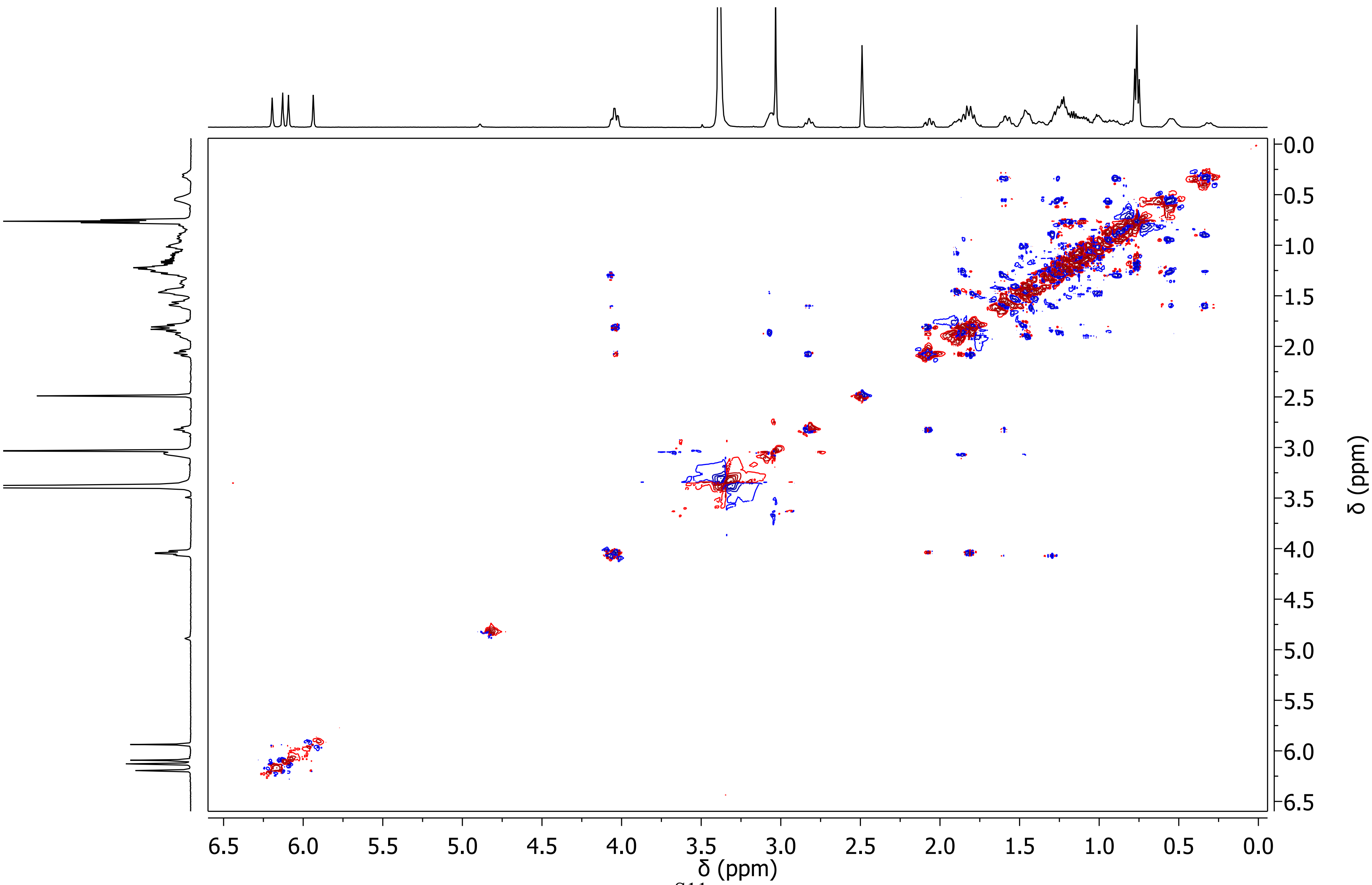

Figure S10. HMBC Spectrum (500 MHz) of **2** in DMSO$_6$

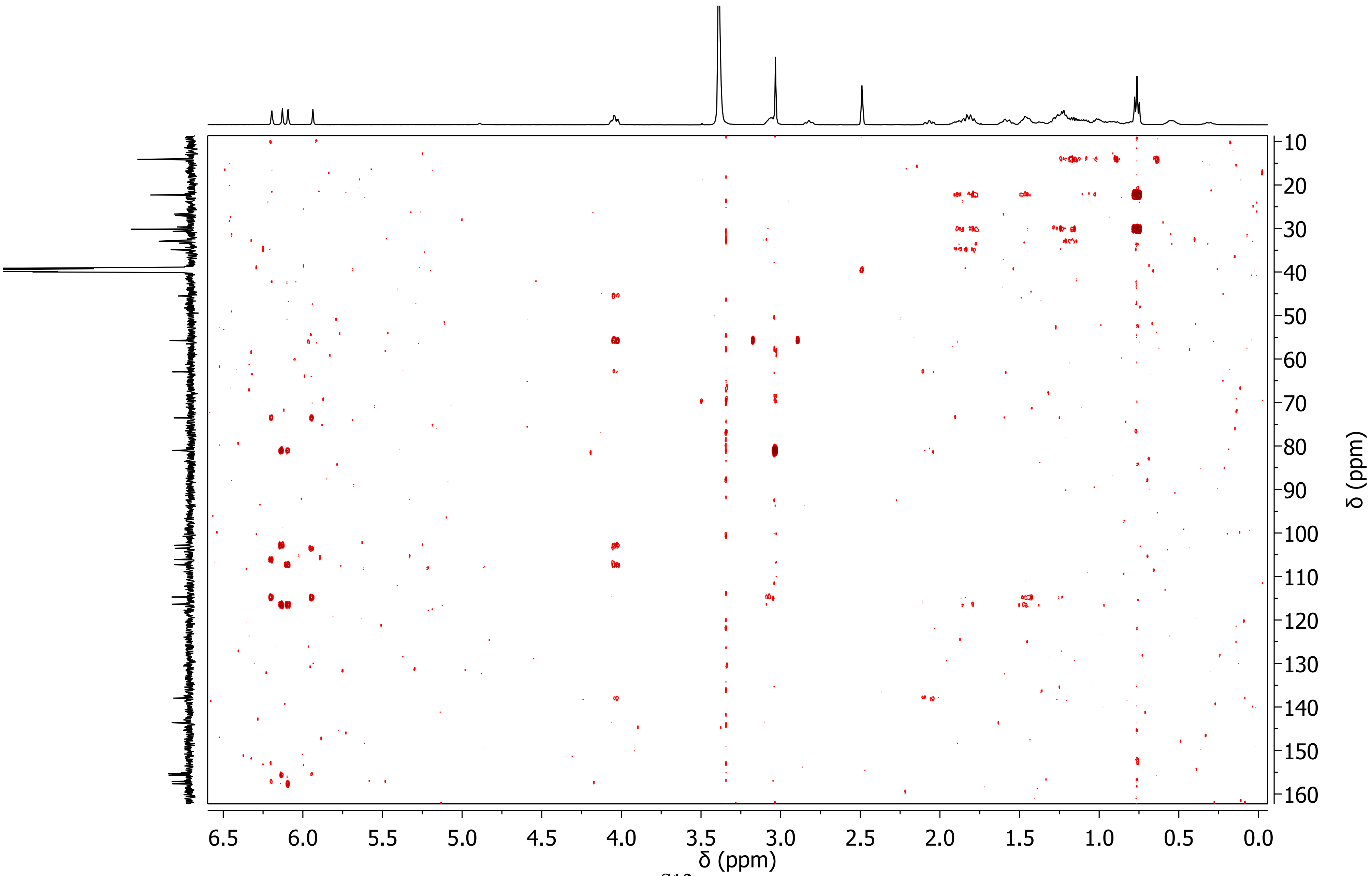

Figure S11. ^{1}H NMR Spectrum (500 MHz) of **3** in DMSO6

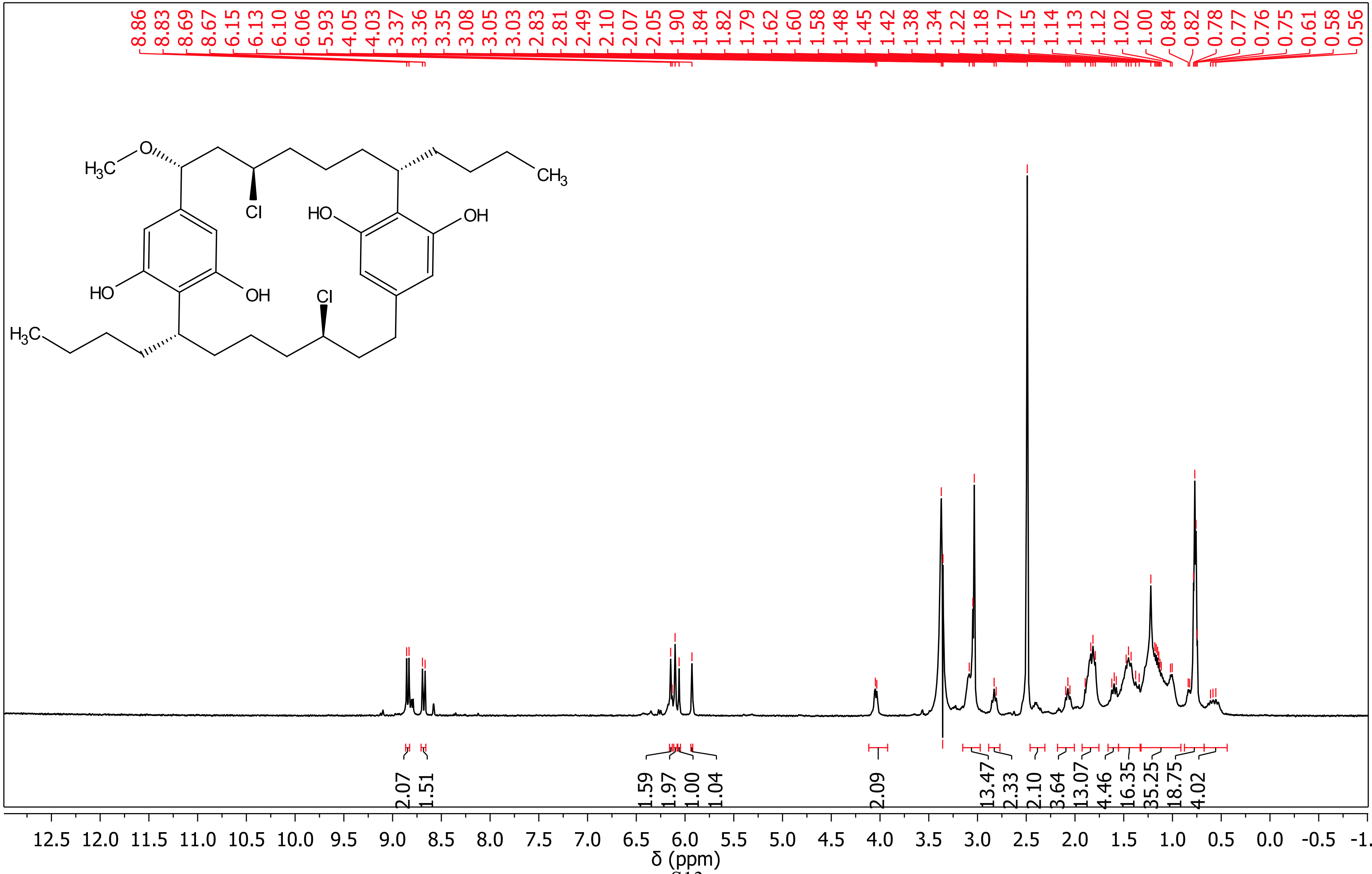

Figure S12. ^{13}C NMR Spectrum (125 MHz) of **3** in DMSO$_6$

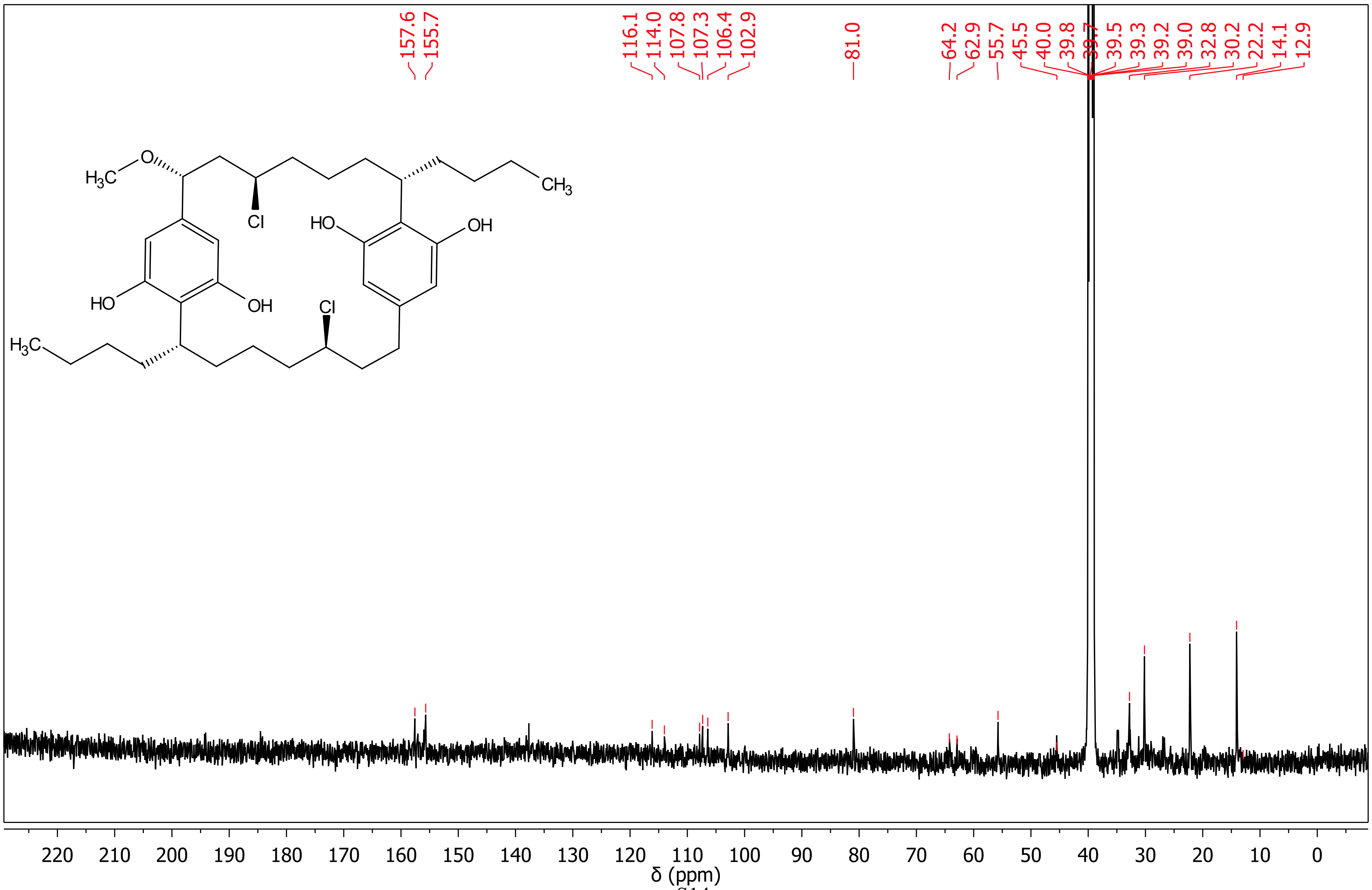

Figure S13. ^{1}H NMR Spectrum (500 MHz) of **4** in $DMSO_6$

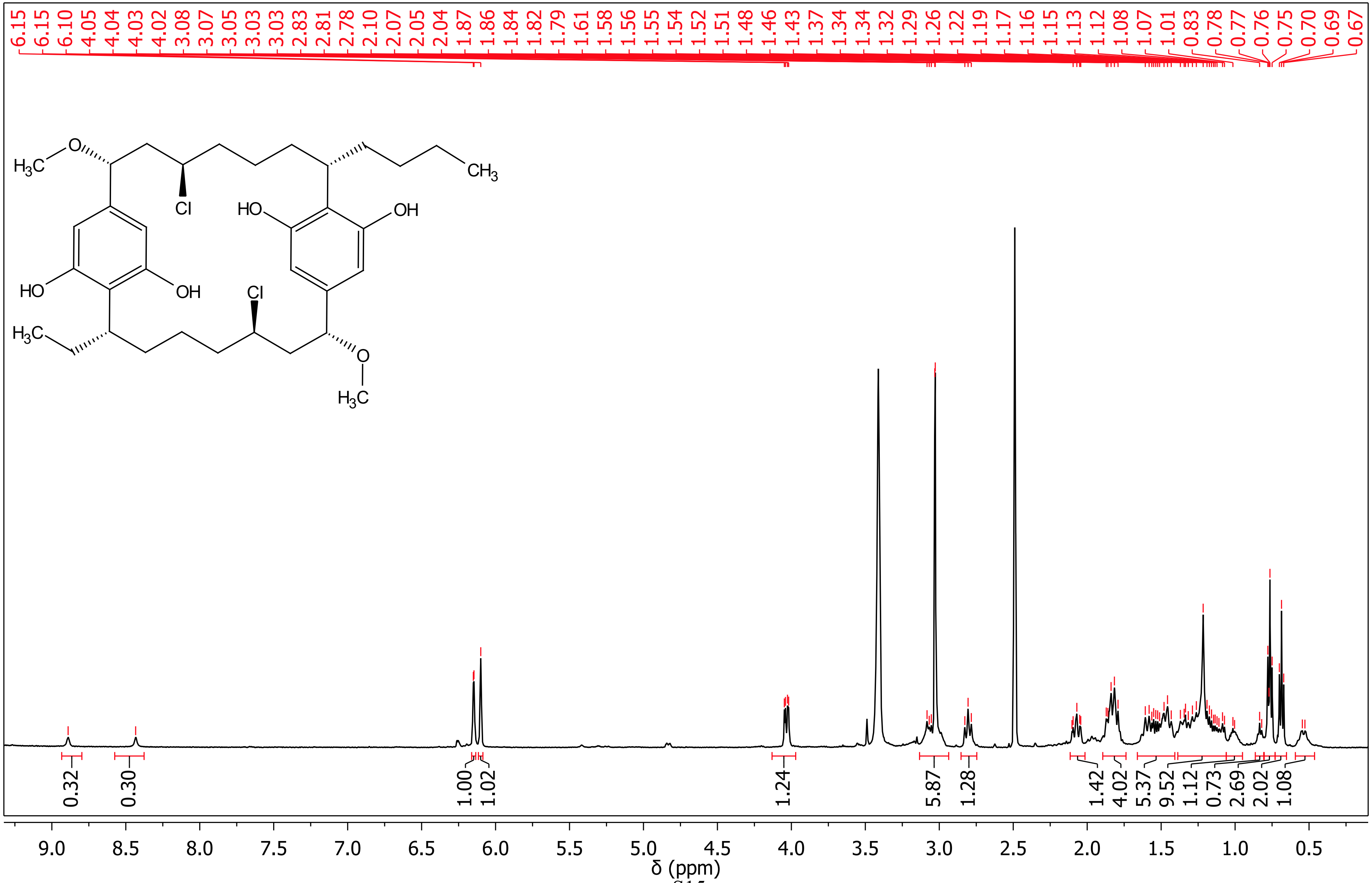

Figure S14. ^{13}C NMR Spectrum (125 MHz) of **4** in DMSO6

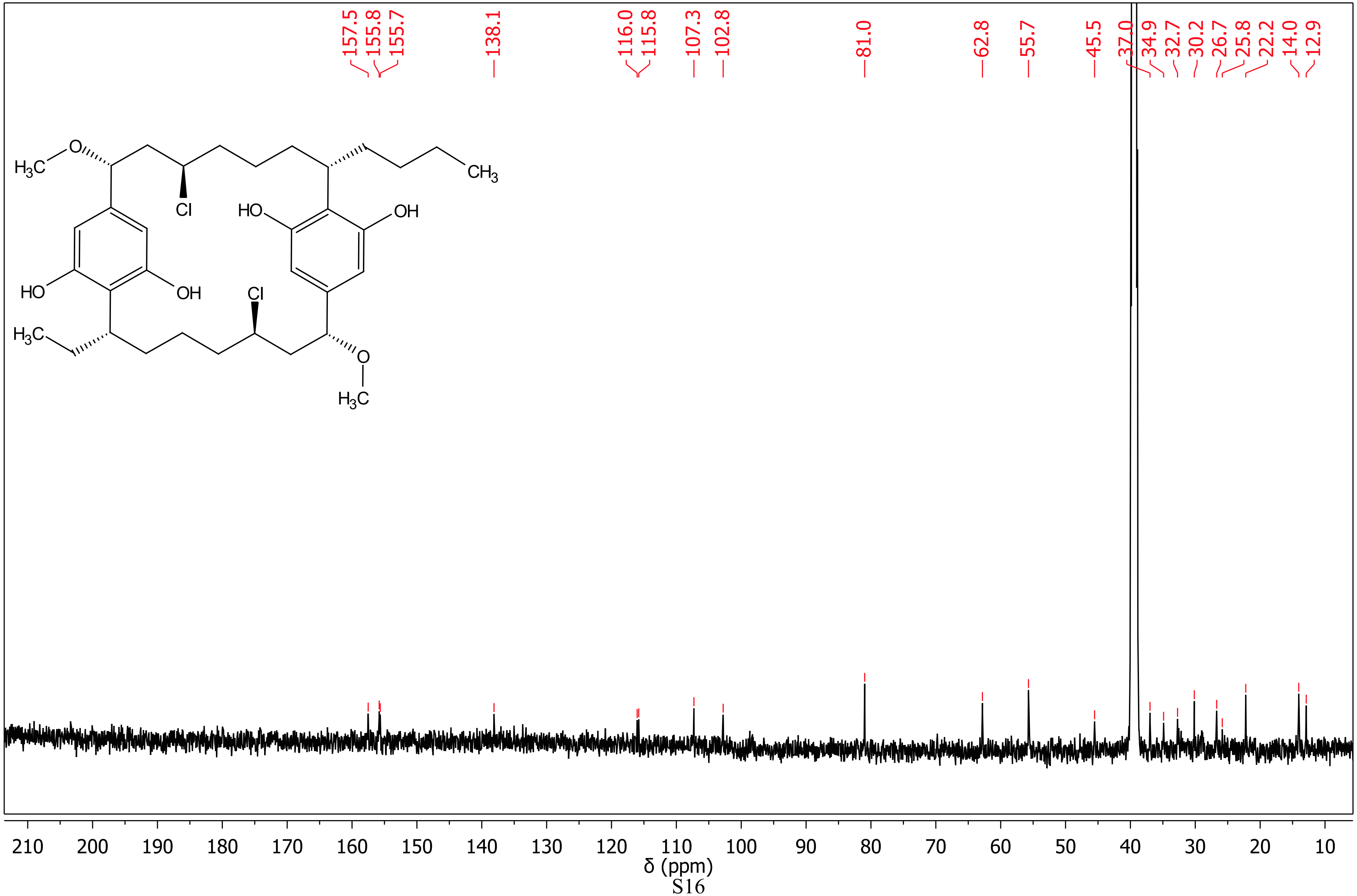

Figure S15. HSQC NMR Spectrum (500 MHz) of **4** in DMSO$_6$

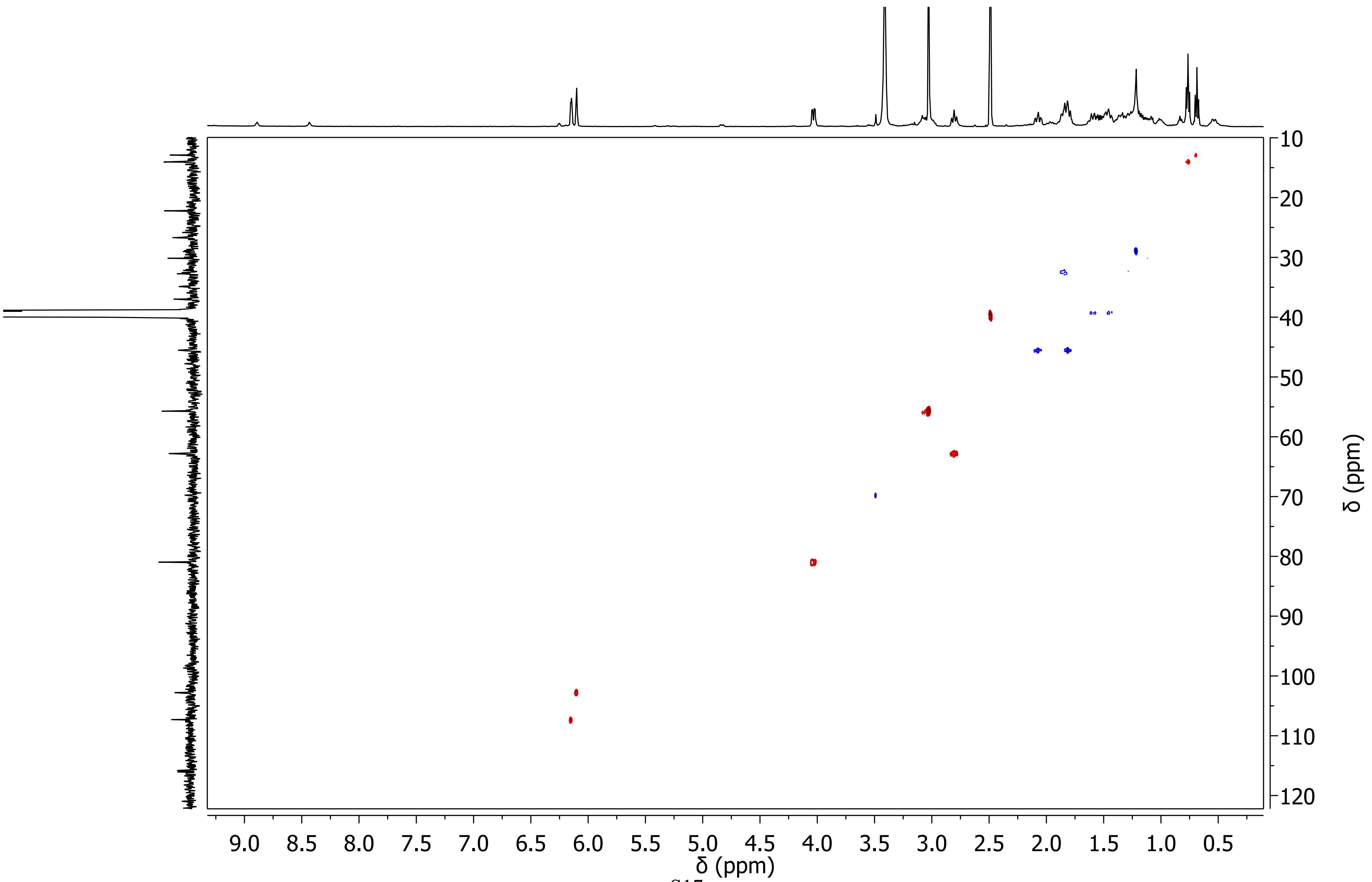

Figure S16. COSY NMR Spectrum (500 MHz) of **4** in DMSO$_6$

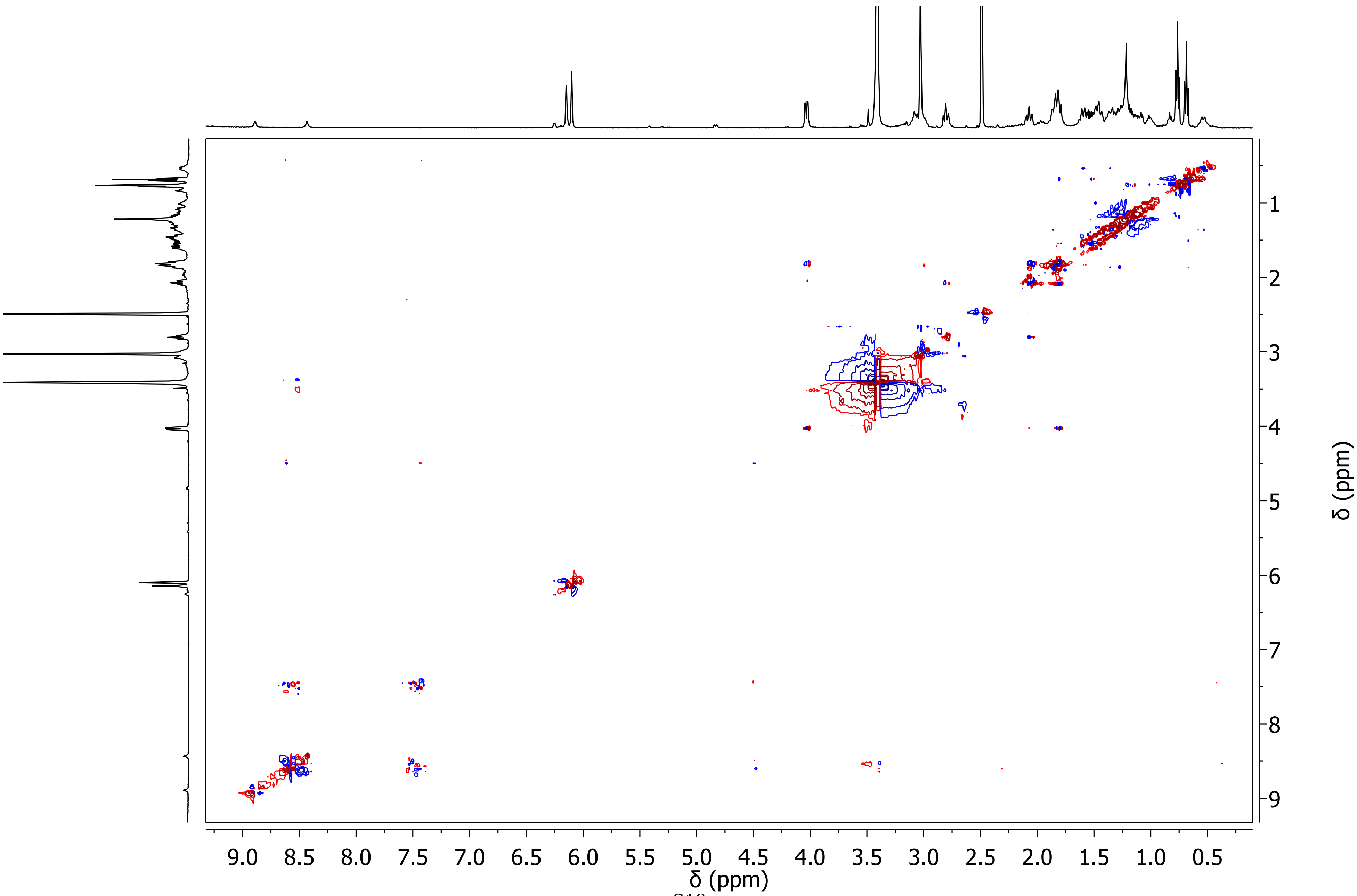

Figure S17. HMBC Spectrum (500 MHz) of **4** in $DMSO_6$

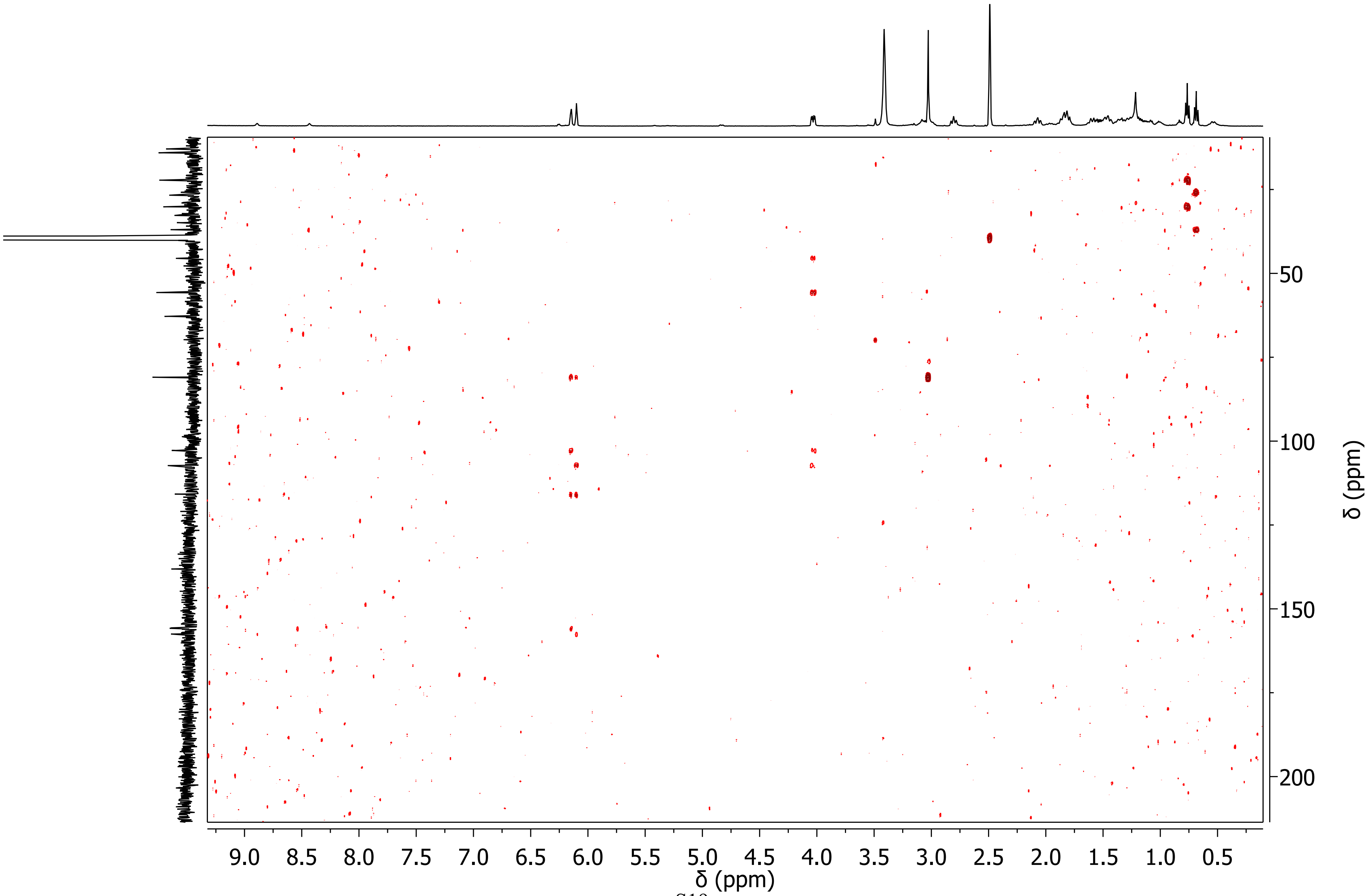

Figure S18. ^{1}H NMR Spectrum (500 MHz) of **5** in $DMSO_6$

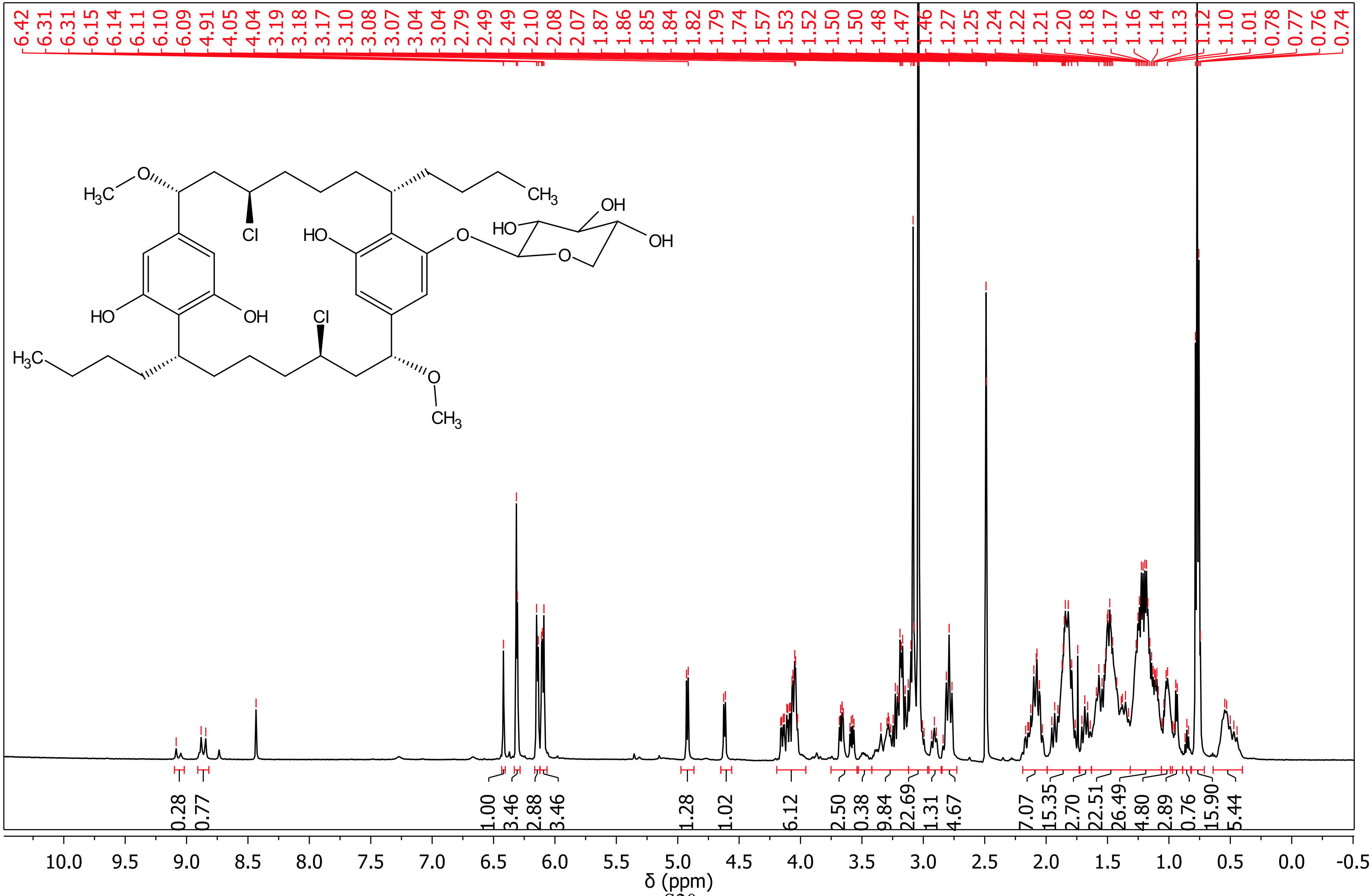

Figure S19. ^{13}C NMR Spectrum (500 MHz) of **5** in DMSO6

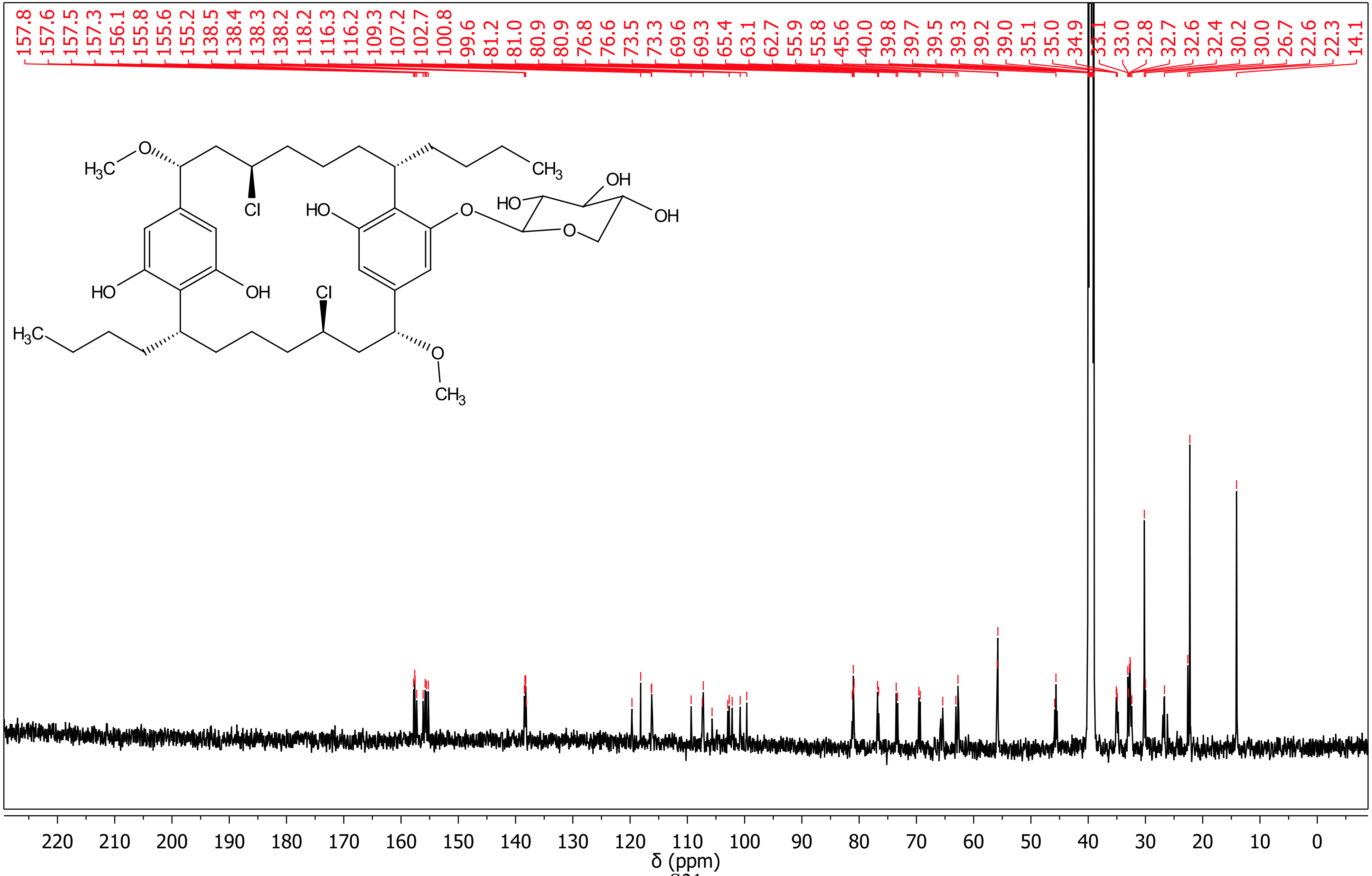

Figure S20. ^{1}H NMR Spectrum (500 MHz) of **6** in $CDCl_3$

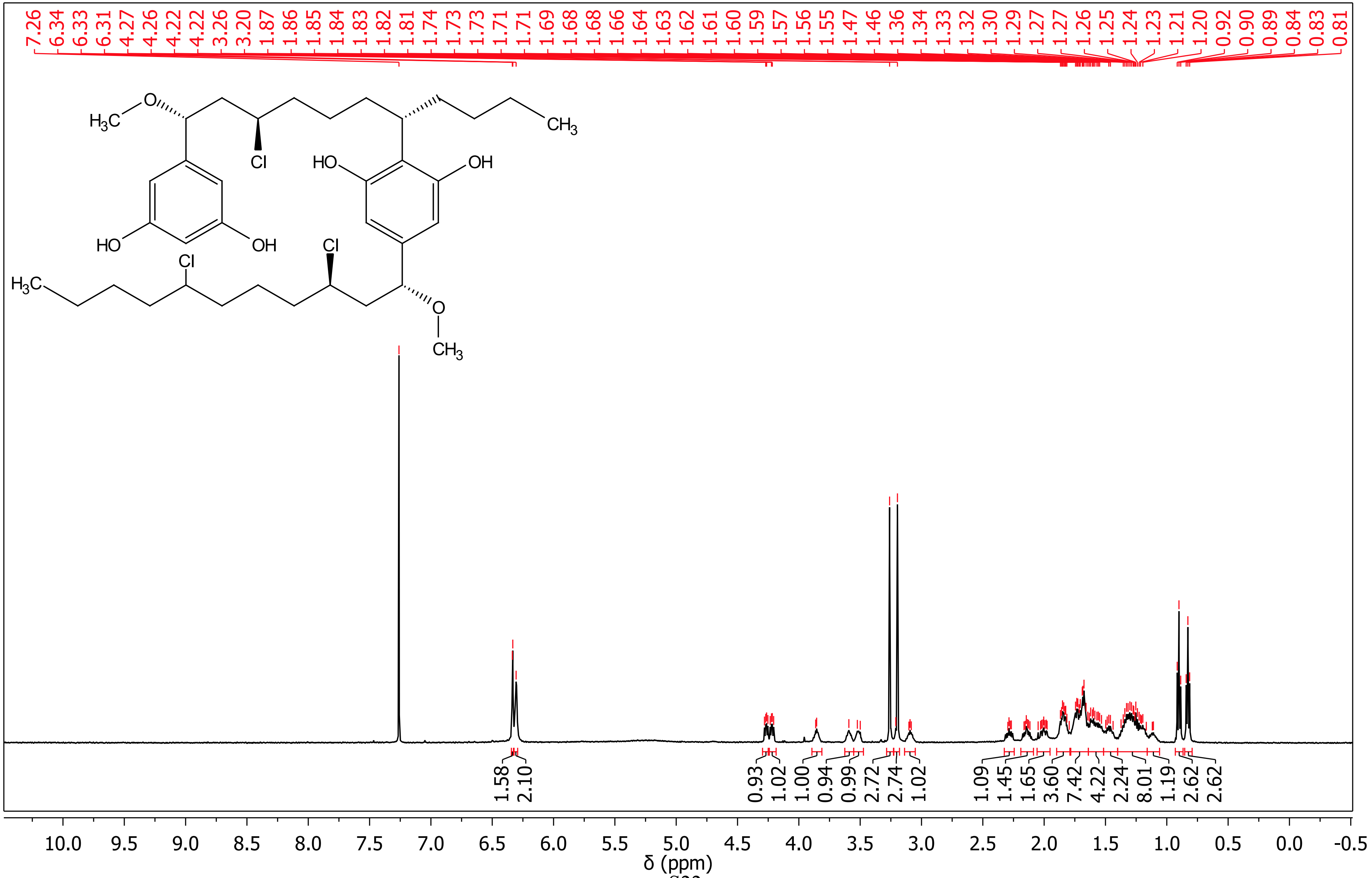

Figure S21. ^{13}C NMR Spectrum (125 MHz) of **6** in $CDCl_3$

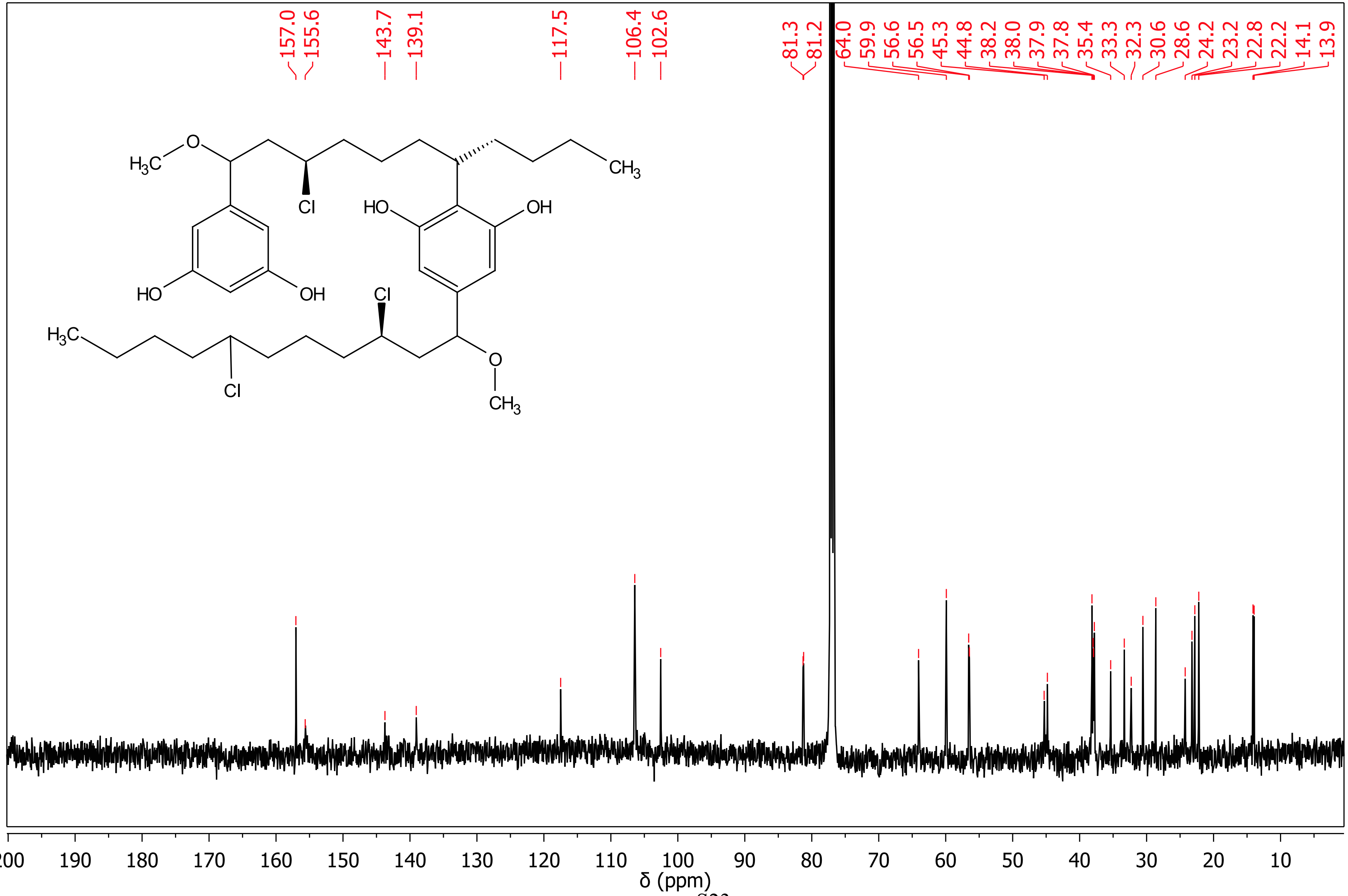

Figure S22. HSQC NMR Spectrum (500 MHz) of **6** in $CDCl_3$

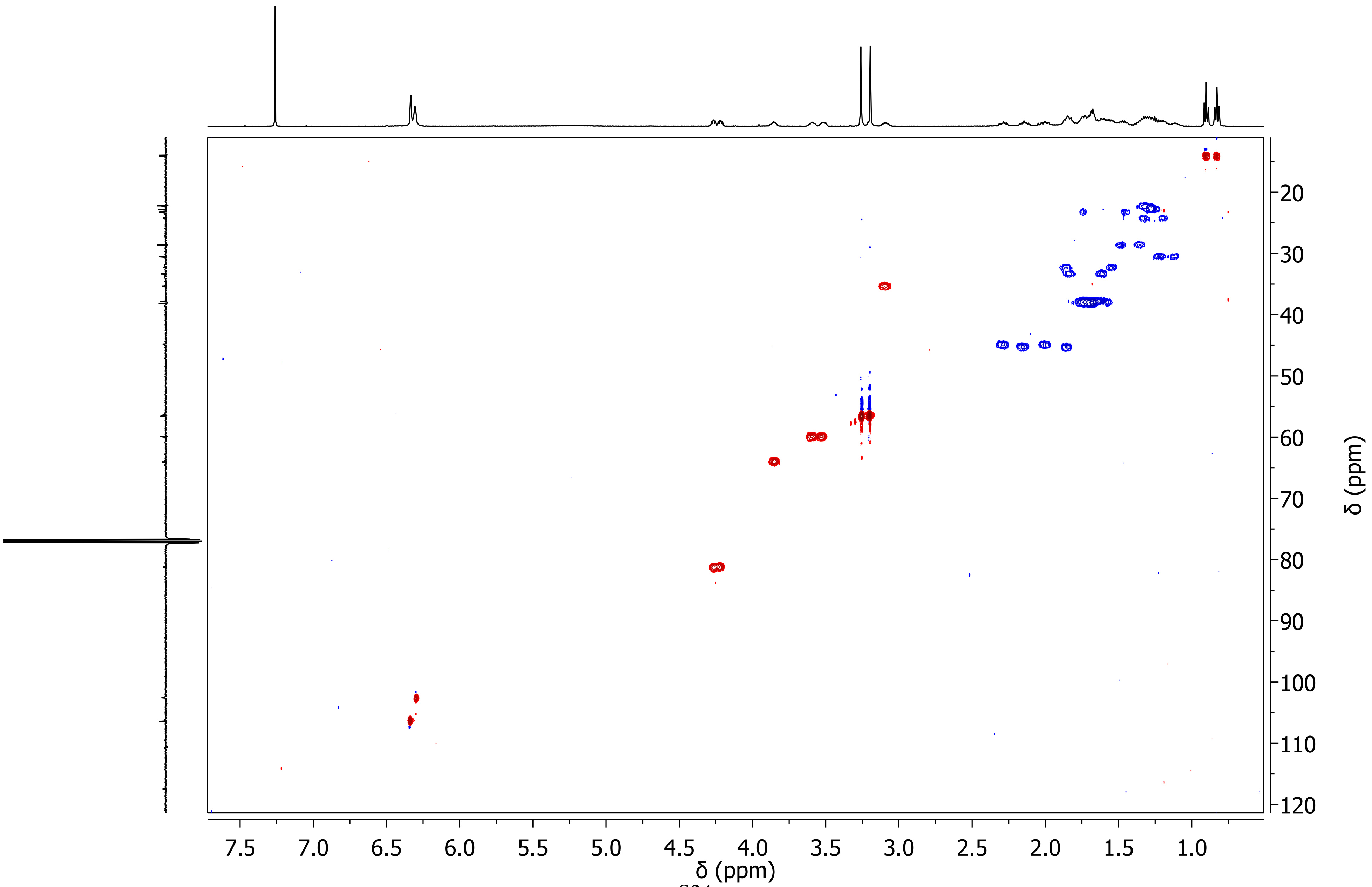

Figure S23. COSY NMR Spectrum (500 MHz) of **6** in $CDCl_3$

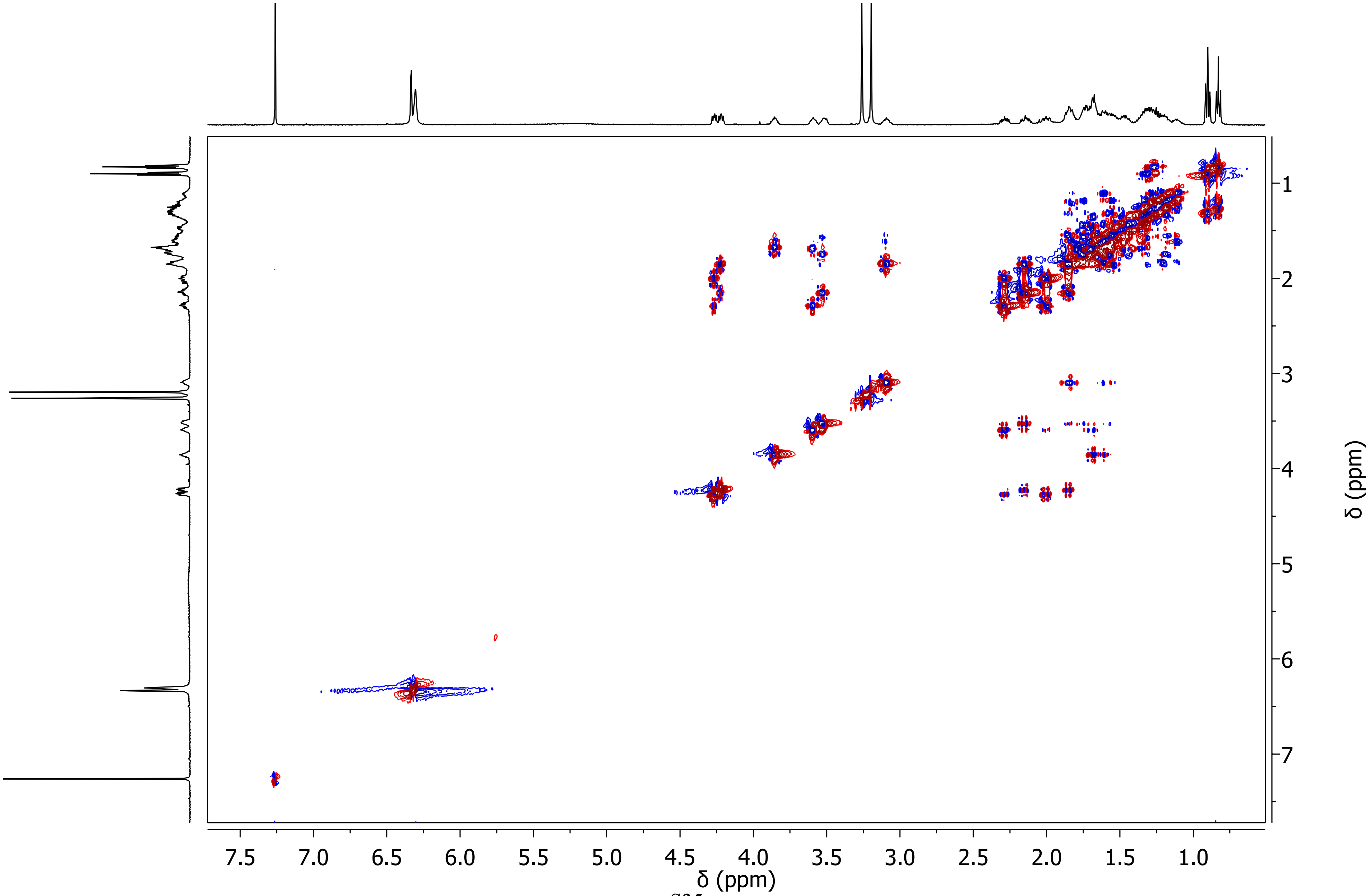

Figure S24. HMBC Spectrum (500 MHz) of **6** in $CDCl_3$

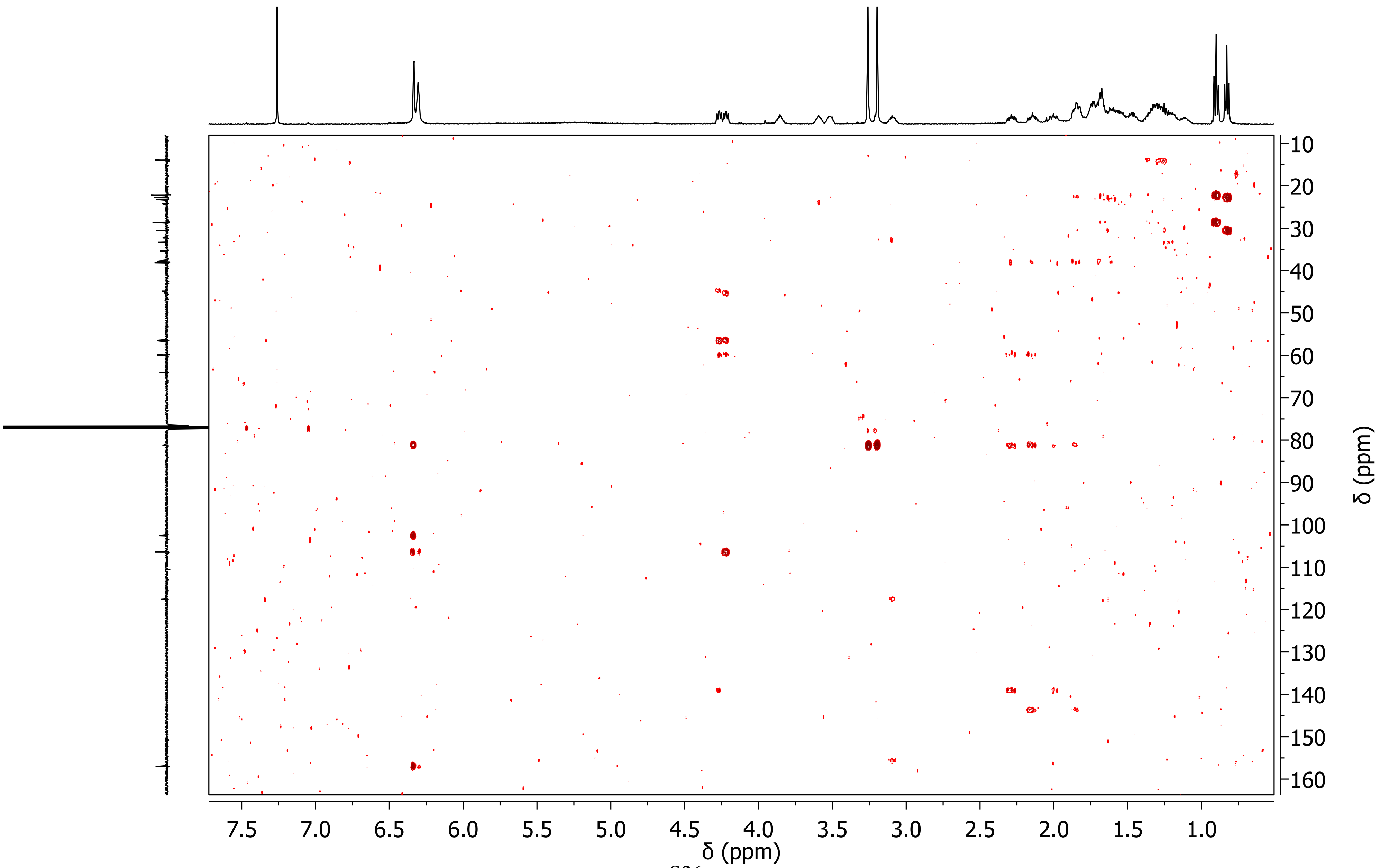

Figure S25. ^{1}H NMR Spectrum (500 MHz) of **6** in DMSO6

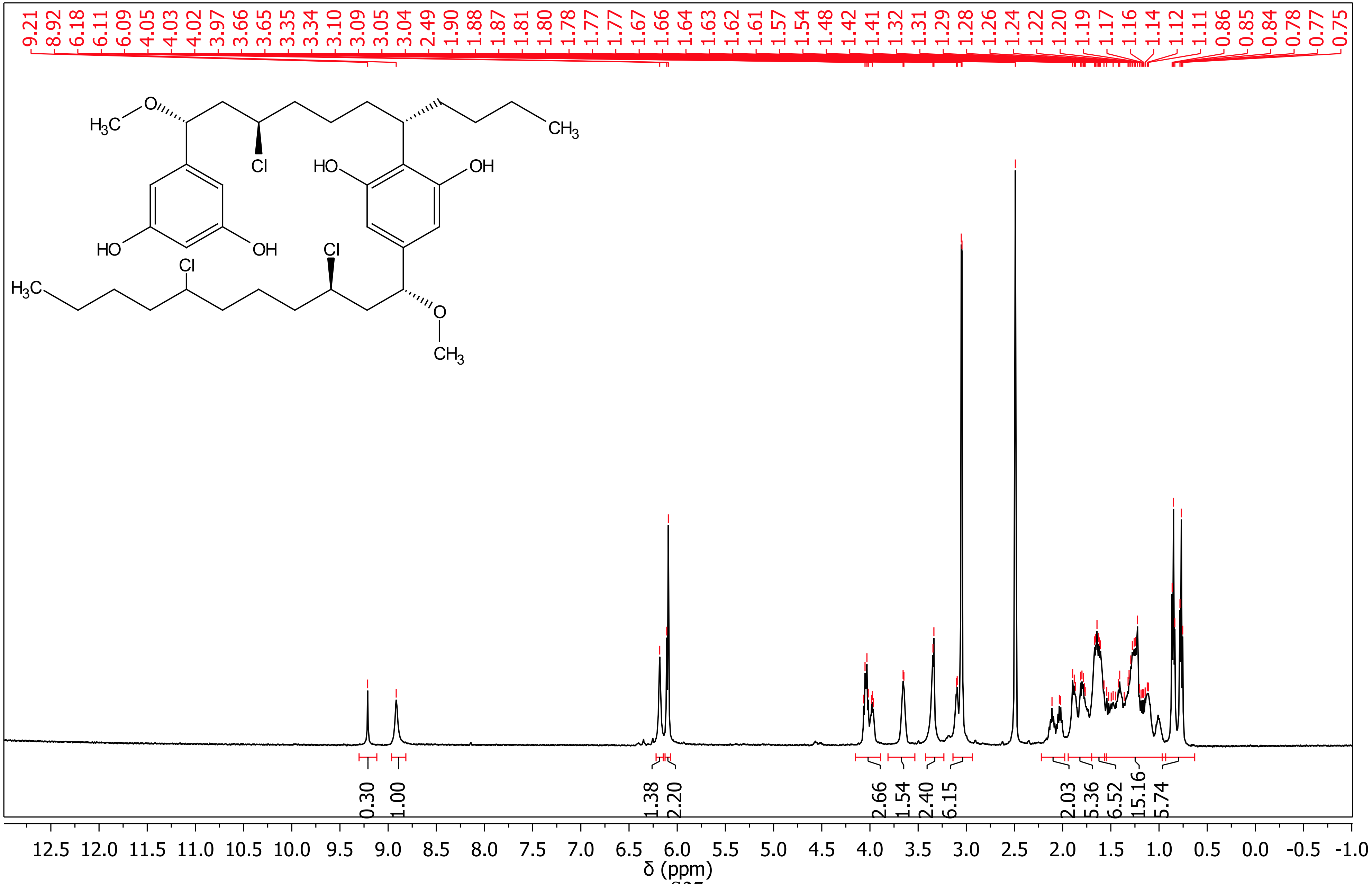

Figure S26. ^{13}C NMR Spectrum (125 MHz) of **6** in DMSO₆

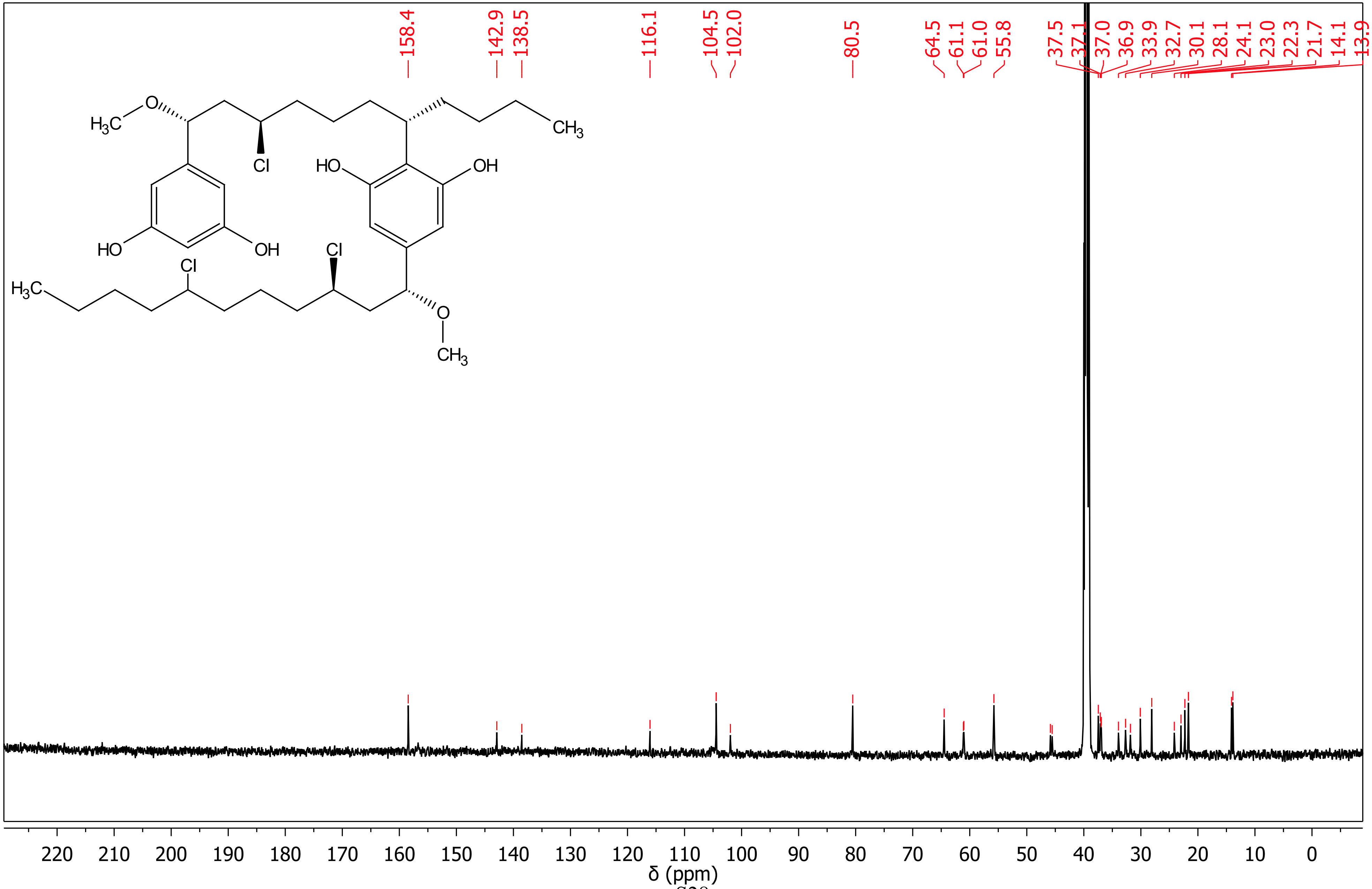

Figure S27. HR-ESI-MS Spectrum of **1**

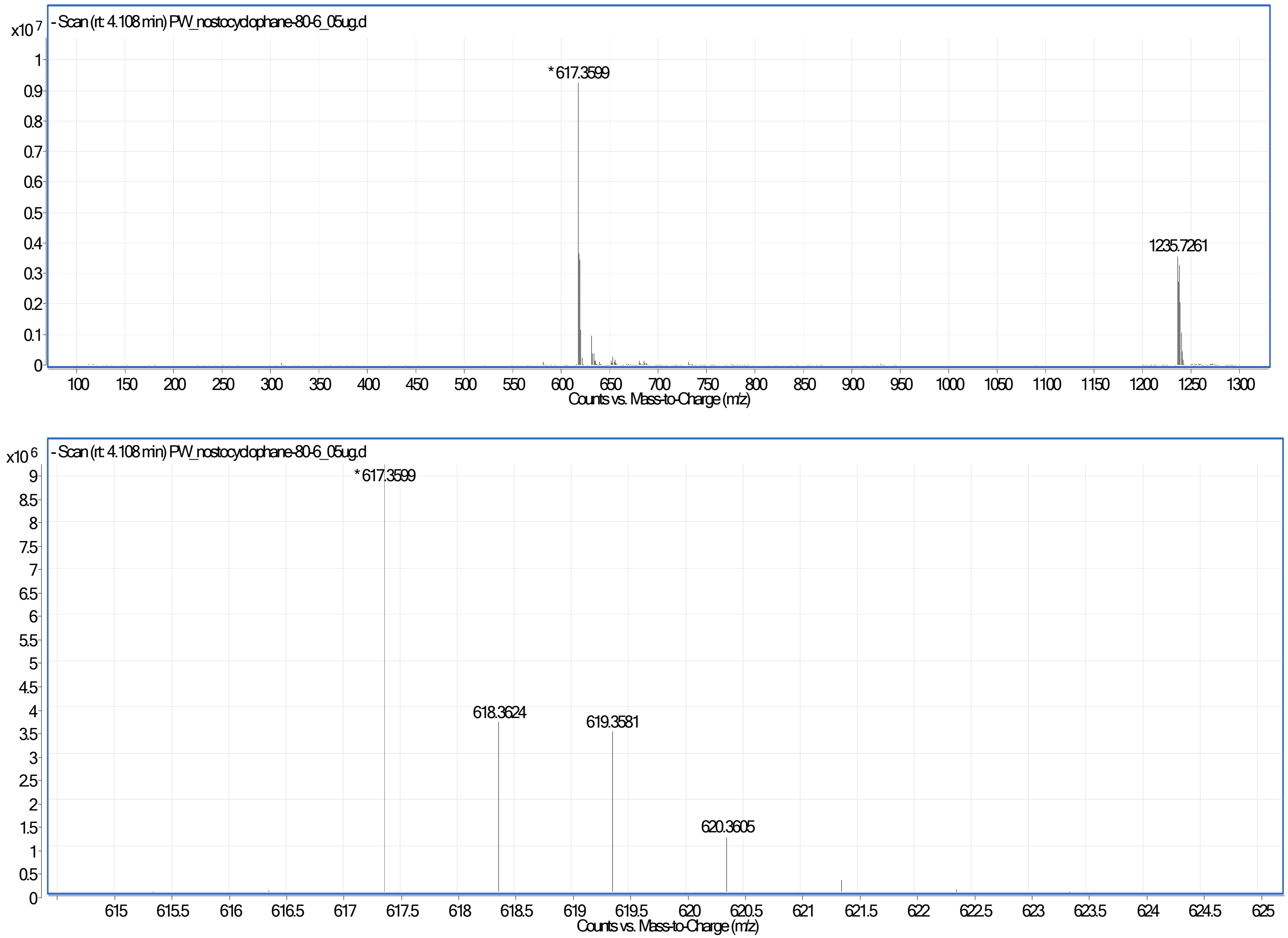

Figure S28. HR-ESI-MS Spectrum of **2**

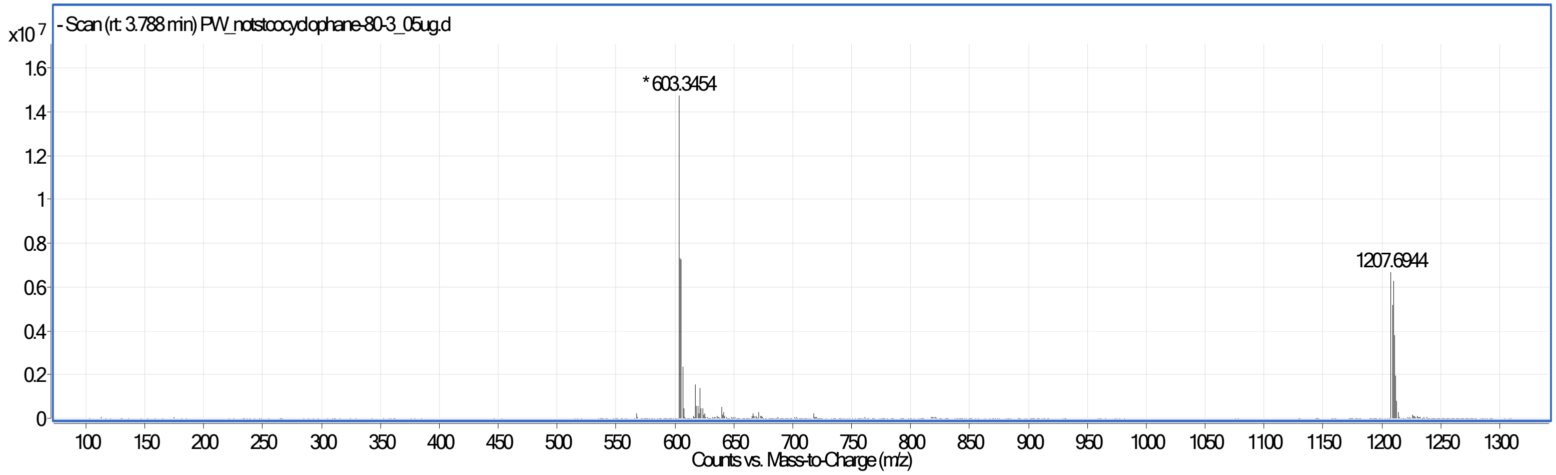

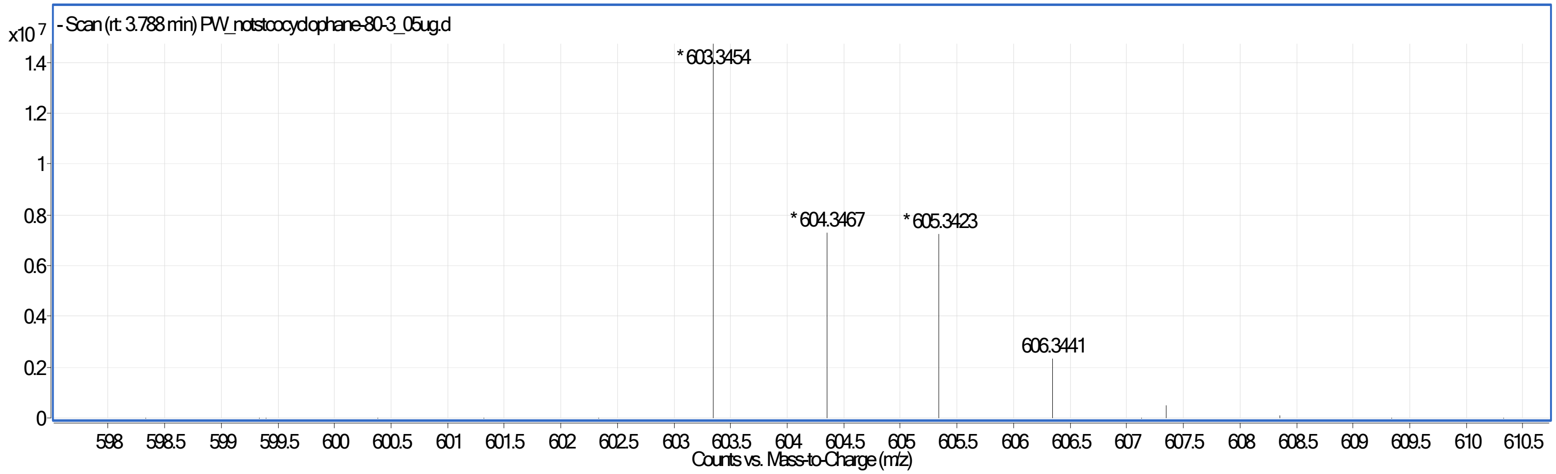

Figure S29. HR-ESI-MS Spectrum of **3**

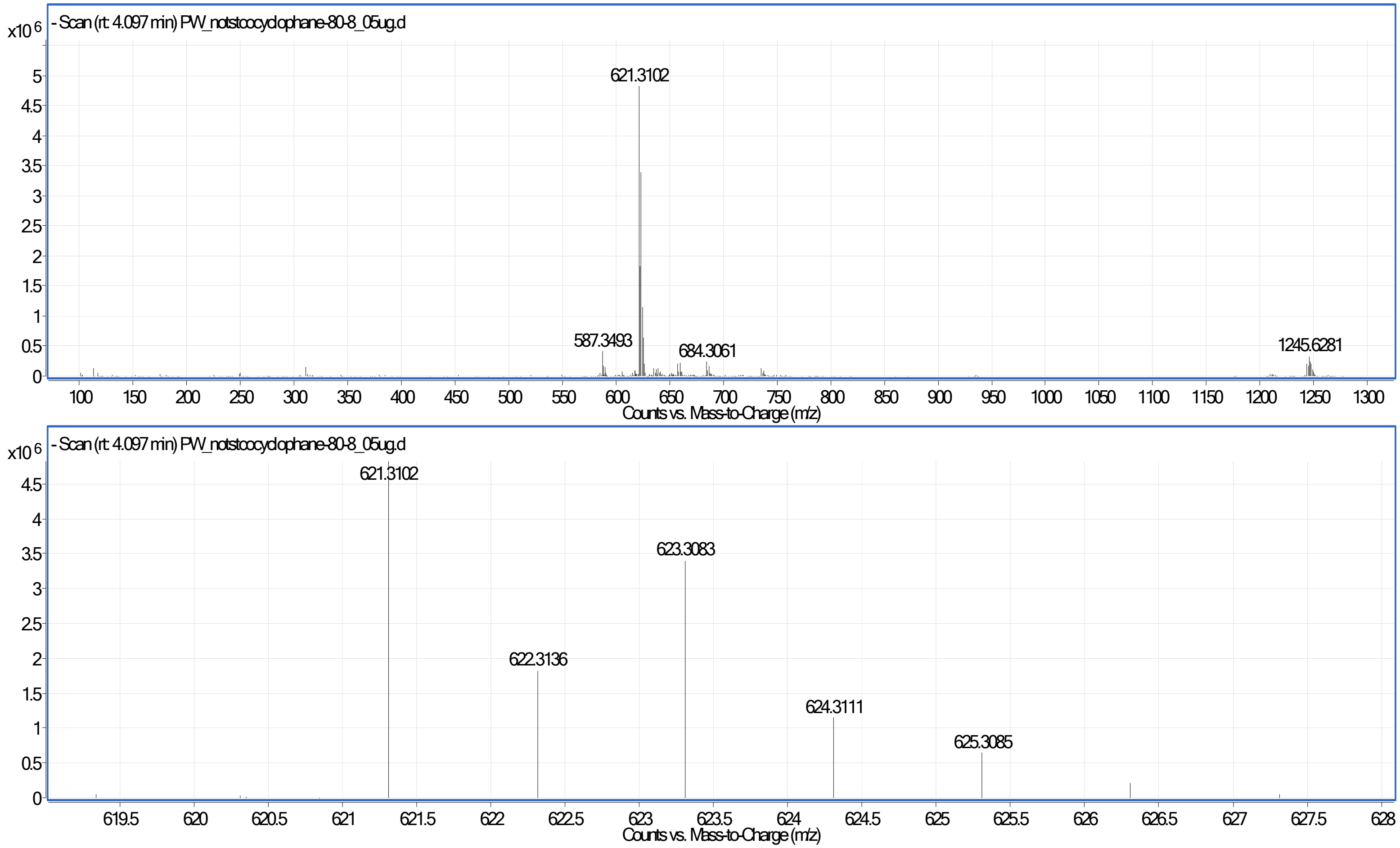

Figure S30. HR-ESI-MS Spectrum of **4**

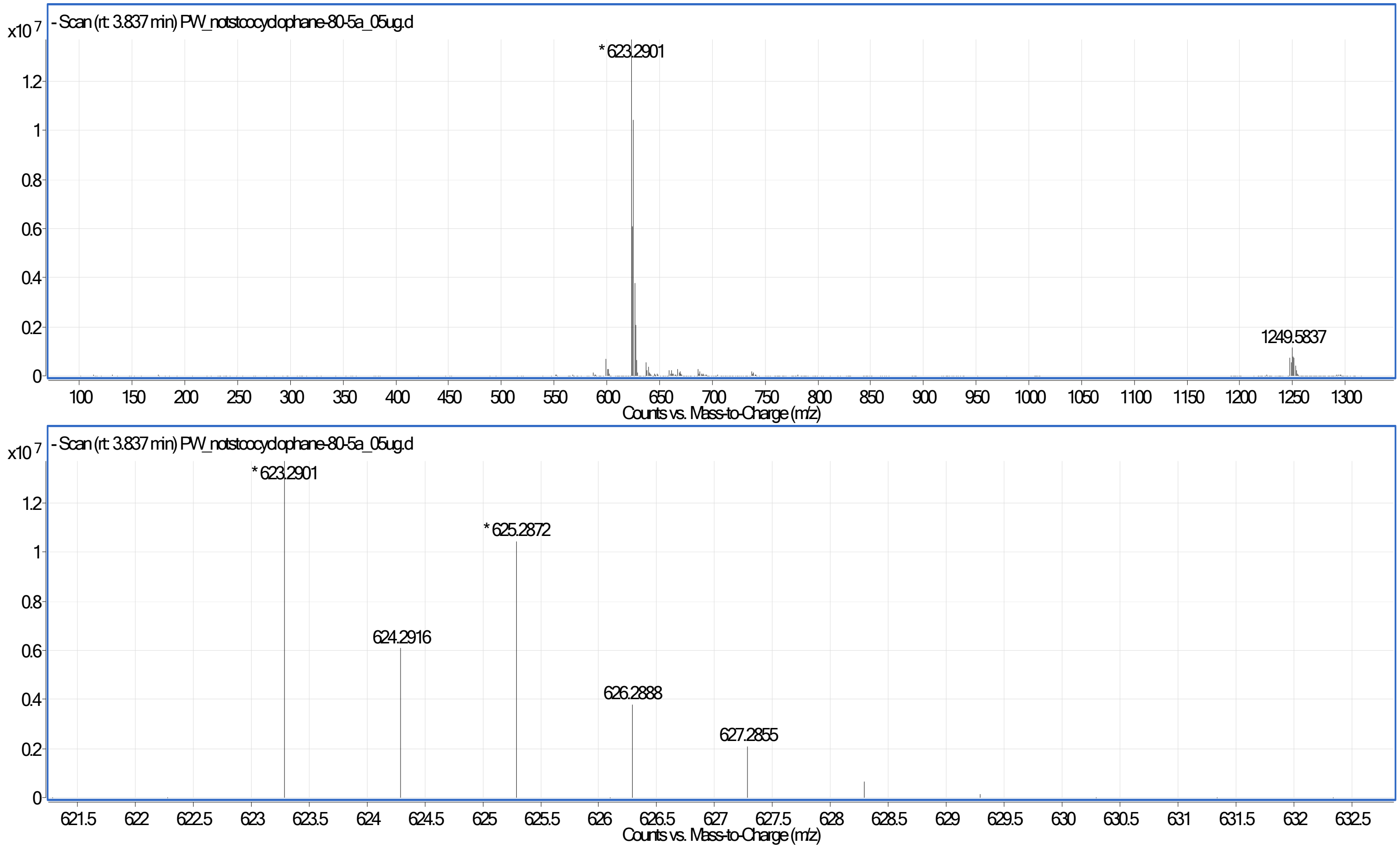

Figure S31. HR-ESI-MS Spectrum of **5**

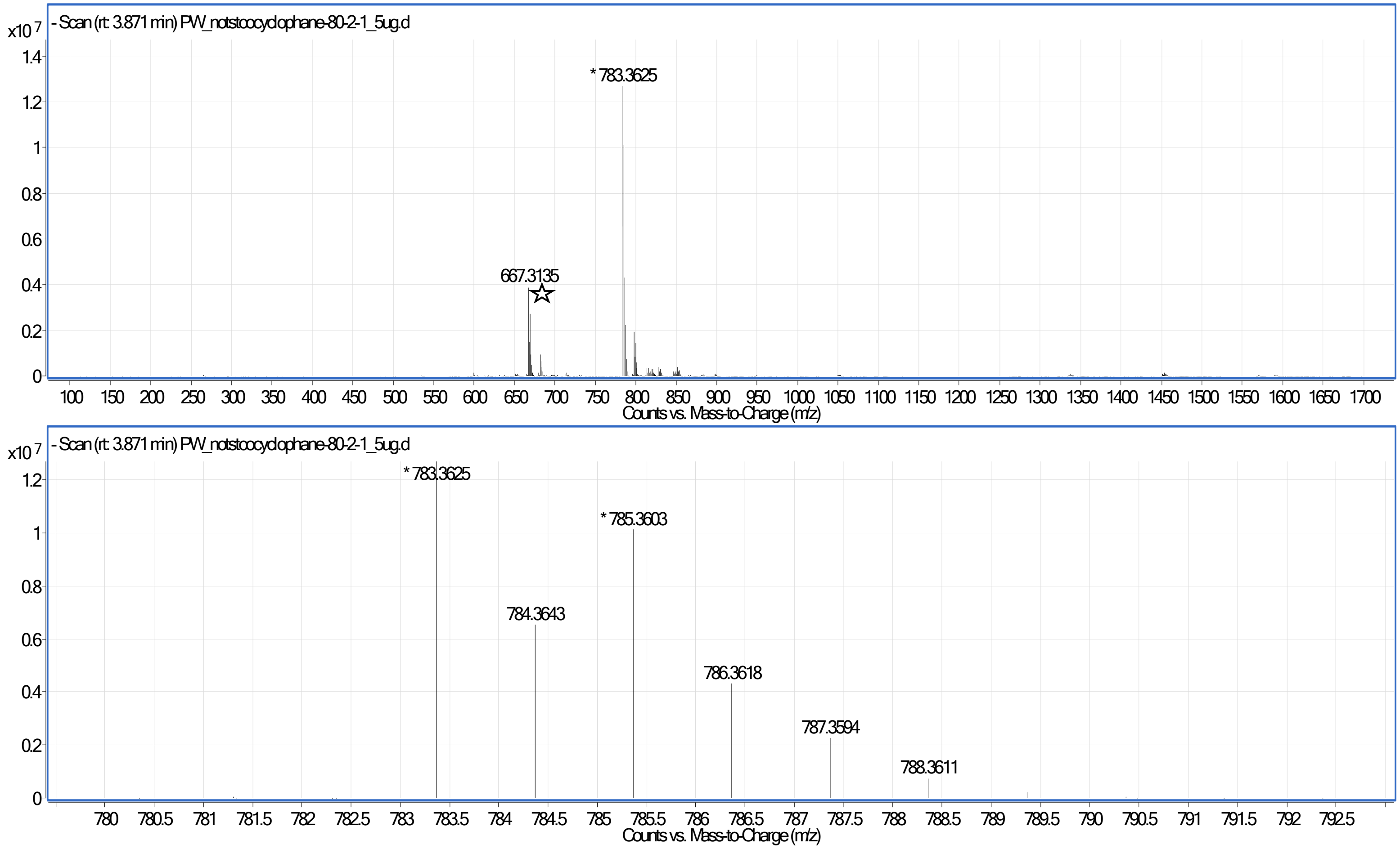

☆ impurity

Figure S32. HR-ESI-MS Spectrum of **6**

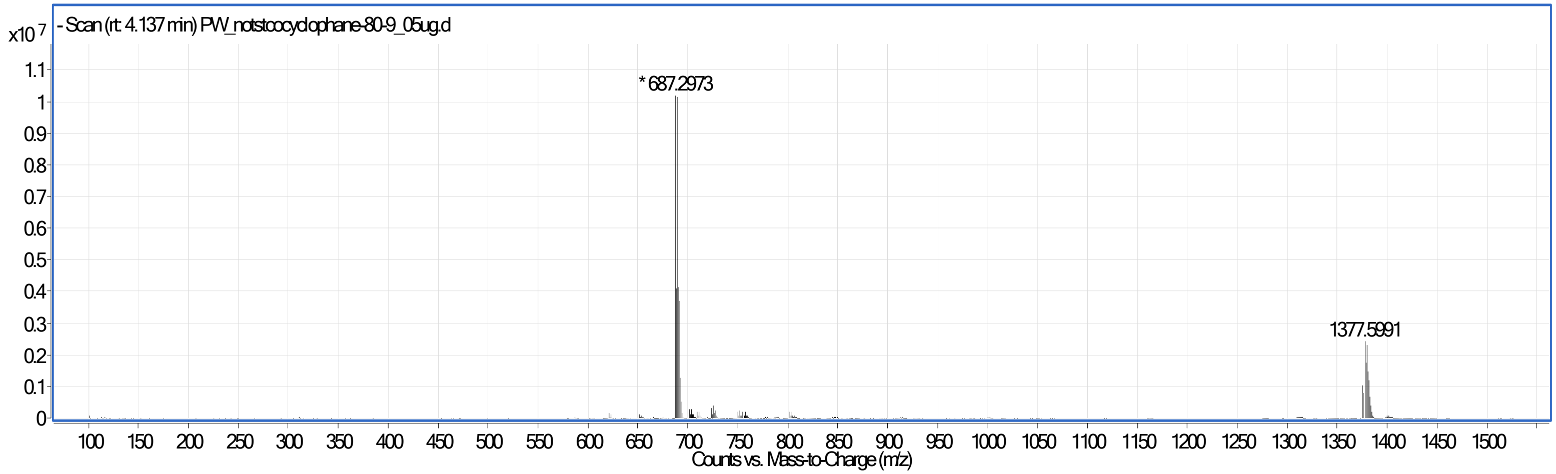

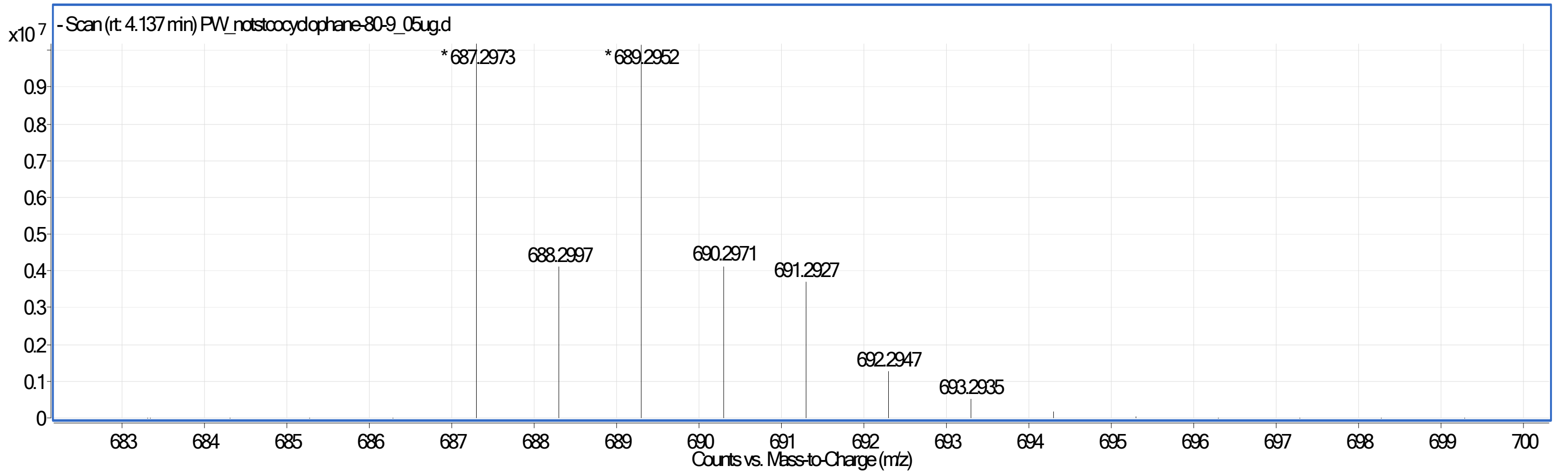

Figure S33. Electronic Circular Dichroism Spectra (MeOH) of Nostocyclophane D, Dedichloronostocyclophane D, and 1-5

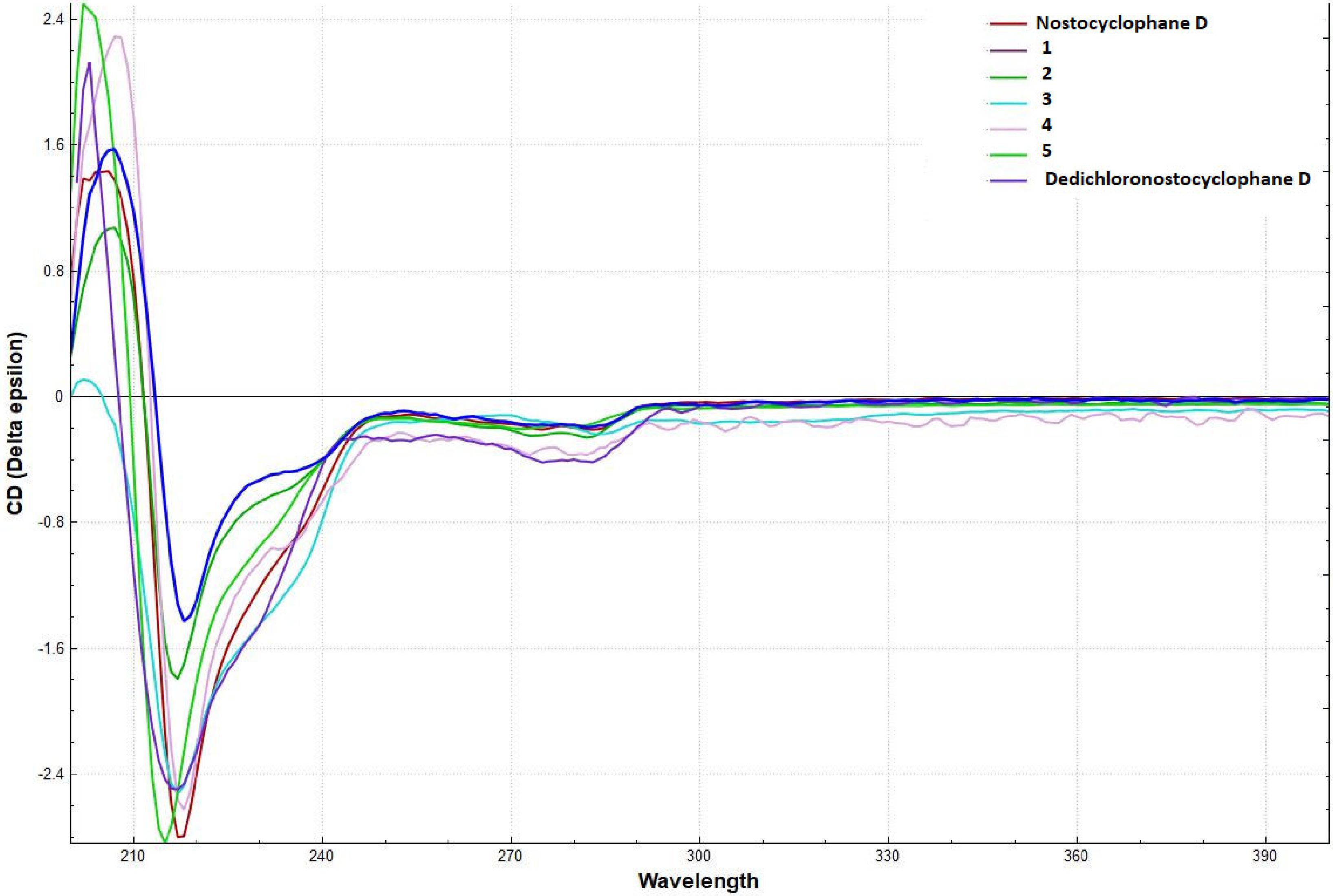

Spectra for compounds 2-5 have been scaled (amplitude increased) to make the comparison easier.

Figure S34. Electronic Circular Dichroism Spectra (MeOH) of 6

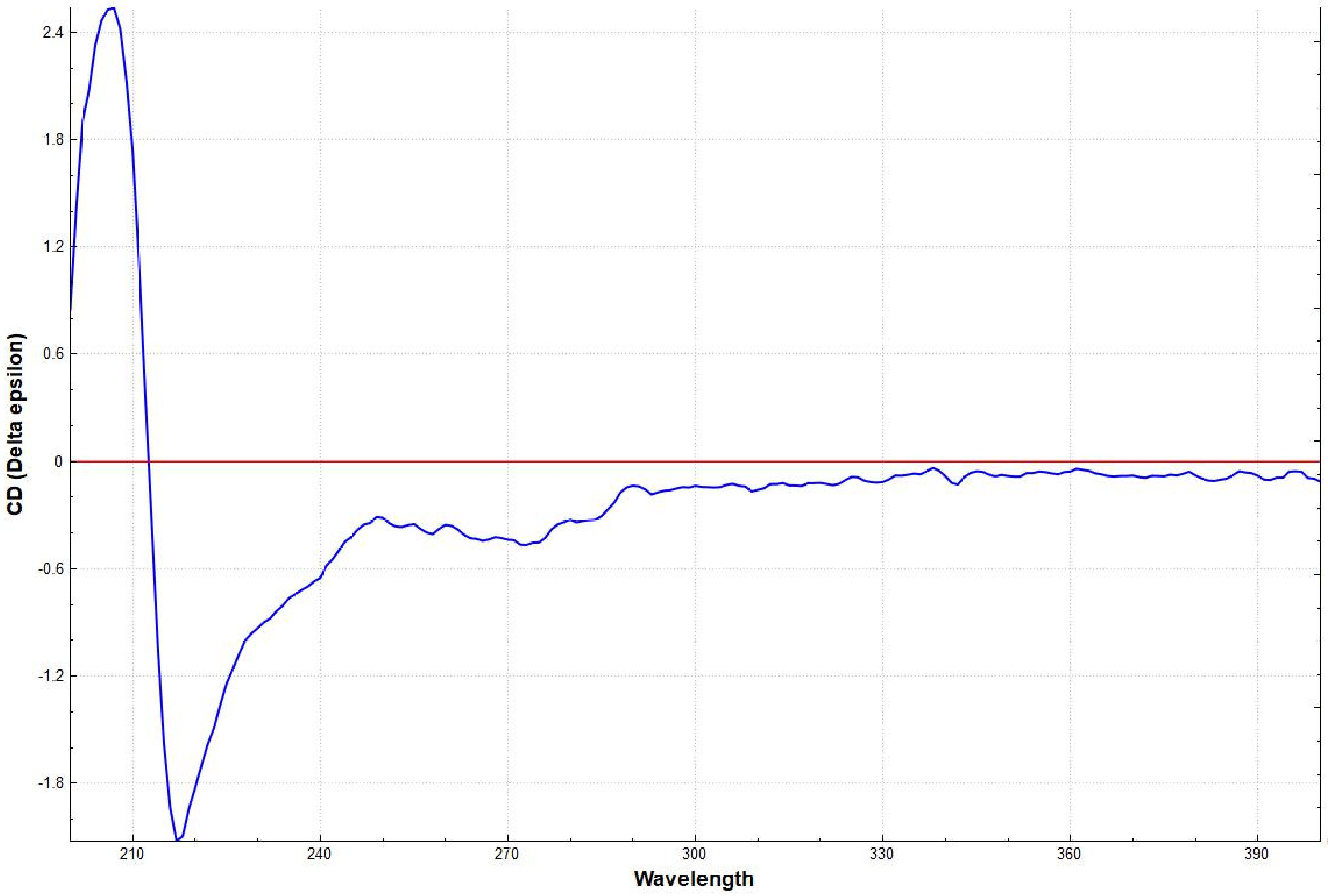

Supplement: Supplementary file 1 [file marinedrugs-21-00101-s001.zip › marinedrugs-1972303-supplementary.pdf]
